# Assessment of Modifiable Factors for the Association of Marital Status With Cancer-Specific Survival

**DOI:** 10.1001/jamanetworkopen.2021.11813

**Published:** 2021-05-28

**Authors:** Zi-Hang Chen, Kai-Bin Yang, Yuan-zhe Zhang, Chen-Fei Wu, Dan-Wan Wen, Jia-Wei Lv, Guang-Li Zhu, Xiao-Jing Du, Lei Chen, Guan-Qun Zhou, Qing Liu, Ying Sun, Jun Ma, Cheng Xu, Li Lin

**Affiliations:** 1 Department of Radiation Oncology, Sun Yat-sen University Cancer Center, State Key Laboratory of Oncology in South China, Collaborative Innovation Center for Cancer Medicine, Guangdong Key Laboratory of Nasopharyngeal Carcinoma Diagnosis and Therapy, Guangzhou, People’s Republic of China; 2 Zhongshan School of Medicine, Sun Yat-sen University, Guangzhou, China; 3 Department of Medical Statistics and Epidemiology, School of Public Health, Sun Yat-sen University, Guangzhou, China

## Abstract

**Importance:**

Married patients with cancer have better cancer-specific survival than unmarried patients. Increasing the early diagnosis and definitive treatment of cancer among unmarried patients may reduce the survival gap.

**Objectives:**

To evaluate the extent to which marriage is associated with cancer-specific survival, stage at diagnosis, and treatment among patients with 9 common solid cancers and to recommend methods for reducing the survival gap.

**Design, Setting, and Participants:**

This retrospective, population-based cohort study included patients older than 18 years who were diagnosed with 1 of 9 common cancers between January 1, 2007, and December 31, 2016. Patient data were retrieved from the Surveillance, Epidemiology, and End Results Program. Statistical analyses were performed from August 1 to October 1, 2020.

**Exposures:**

Marital status, classified as married and unmarried (including single, separated, divorced, widowed, and unmarried patients or domestic partners).

**Main Outcomes and Measures:**

The primary outcome was the time ratio (TR) of cancer-specific survival (married vs unmarried). Mediation analyses were conducted to determine the extent to which the association of marriage with cancer-specific survival was mediated by stage at diagnosis and treatment.

**Results:**

This study included 1 733 906 patients (894 379 [51.6%] women; 1 067 726 [61.6%] married; mean [SD] age, 63.76 [12.60] years). Multivariate analyses found that those who were married were associated with better cancer-specific survival than unmarried patients (TR, 1.36; 95% CI, 1.35-1.37). Early diagnosis in breast cancer, colorectal cancer, endometrial cancer, and melanoma mediated the association between marital status and cancer-specific survival (breast cancer: proportion mediated [PM], 11.4%; 95% CI, 11.2%-11.6%; colorectal cancer: PM, 10.9%; 95% CI, 10.7%-11.2%; endometrial cancer: PM, 12.9%; 95% CI, 12.5%-13.3%; melanoma: PM, 12.0%; 95% CI, 11.7-12.4%). Surgery mediated the association between marital status and cancer-specific survival in lung (PM, 52.2%; 95% CI, 51.9%-52.4%), pancreatic (PM, 28.9%; 95% CI, 28.6%-29.3%), and prostate (PM, 39.3%; 95% CI, 39.0%-39.6%) cancers. Chemotherapy mediated the association of marital status with cancer-specific survival in lung (PM, 37.7%; 95% CI, 37.6%-37.9%) and pancreatic (PM, 28.6%; 95% CI, 28.4%-28.9%) cancers. Improved cancer-specific survival associated with marriage was greater among men than women (men: TR, 1.27; 95% CI, 1.25-1.28; women: TR, 1.20; 95% CI, 1.19-1.21). The contribution of receiving an early diagnosis and treatment with surgery or chemotherapy to the association between marital status and cancer-specific survival was greater among men than women (early diagnosis: PM, 21.7% [95% CI, 21.5%-21.9%] vs PM, 20.3% [95% CI, 20.2%-20.4%]; surgery: PM, 26.6% [95% CI, 26.4%-26.7%] vs PM, 11.1% [95% CI, 11.0%-11.2%]; chemotherapy: PM, 6.8% [95% CI, 6.7%-6.8%] vs PM, 5.1% [95% CI, 5.0%-5.2%]).

**Conclusions and Relevance:**

In this study, survival disparities associated with marital status were attributable to early diagnosis in breast, colorectal, and endometrial cancers as well as melanoma and to treatment-related variables in lung, pancreatic, and prostate cancers. The findings also suggest that marriage may play a greater protective role in the cancer-specific survival of men than of women.

## Key Points

**Question:**

What are the associations of cancer stage at time of diagnosis, treatment, and marital status with cancer-specific survival?

**Findings:**

This cohort study of 1.7 million adults with cancer found that stage at diagnosis mediated the association of marriage with cancer-specific survival in breast, colorectal, and endometrial cancers as well as melanoma; receiving surgery mediated the association in lung, pancreatic, and prostate cancers; and receiving chemotherapy mediated the association in lung and pancreatic cancers.

**Meaning:**

These findings suggest that promoting the early diagnosis of breast, colorectal, and endometrial cancers and melanoma and providing definitive treatment for lung, pancreatic, and prostate cancers could improve cancer-specific survival among unmarried patients with cancer.

## Teaser

This cohort study evaluates the extent to which marriage is associated with cancer-specific survival, stage at diagnosis, and treatment among patients with 9 common solid cancers and recommends methods for reducing the survival gap.

## Introduction

Cancer has the largest disease burden worldwide and is the second leading cause of death globally^1^ and in the United States.^2^ Unmarried patients with cancer (including those who have never married, are separated, divorced, widowed, or in a domestic partnership) are at a higher risk of dying from cancer than married patients.^3,4,5,6,7^ In the United States, the proportion of unmarried people is increasing; in 2020, 50% of US residents were married, and this percentage has decreased 9 percentage points during the past 25 years.^8^ In recent decades, the US marriage rate has decreased, from a rate of 9.8 marriages per 1000 people in 1990 to a rate of 6.5 per 1000 people in 2018, reaching a historic low.^9^ As unmarried patients with cancer have poorer cancer-specific survival (CSS) and their proportion is steadily increasing, details of the mechanisms underlying the association between marital status and CSS should be explored, as they may inform specific interventions for unmarried patients that will improve their CSS.

Unmarried patients with cancer have a higher risk of being diagnosed with cancer at an advanced stage of the disease than married patients.^3,7,10,11,12,13^ Moreover, studies have revealed that unmarried patients are less likely to receive appropriate treatment than married patients.^3,7,13,14^ The association between being married and better CSS has been speculated to be secondary to the benefits of early diagnosis and treatment.^3,13,15,16^ However, no studies have confirmed whether or to what extent marital status is associated with CSS by its association with the stage of cancer at diagnosis or the treatment received. Few population-based studies have compared the association between marriage and CSS in men with that in women; hence, a thorough analysis is needed.

Mediation analysis is a useful approach to quantify the importance of pathways by which an exposure variable is associated with an outcome variable.^17^ The objective of this study was to conduct a mediation analysis to assess whether stage at diagnosis or treatment mediates the association between marital status and CSS and to determine quantitatively their proportion of mediation on 9 common solid cancers using the Surveillance, Epidemiology, and End Results (SEER) database. Sex differences in the association of marriage with CSS were also examined.

## Methods

### Study Cohort

We used the SEER Database from 1975 to 2016, which includes statistics from 1975 through 2016, was based on the November 2018 SEER data submission, and was posted to the SEER website in April 2019. Data for 2 131 538 patients diagnosed from 2007 through 2016 with 1 of 9 common solid cancers (ie, breast, lung, prostate, colorectal, bladder, kidney, endometrial, and pancreatic cancers and melanoma) were retrieved from the SEER database.^2^ Patients were excluded if information about their marital status (n = 263 147 [12.3%]), cancer stage at diagnosis (n = 108 440 [5.1%]), surgery (n = 15 531 [0.7%]), or cause of death (n = 7565 [0.4%]) was unavailable or if they were younger than 18 years at diagnosis (n = 2949 [0.1%]), leaving 1 733 906 patients in the final cohort. Because this data set is in the public domain, it was exempt from institutional review board approval and the requirement for informed consent, per the Common Rule (45 CFR part 46). This study followed the Strengthening the Reporting of Observational Studies in Epidemiology (STROBE) reporting guideline.

### Study Variables

In the SEER database, marital status included the categories married (including common law), single (never married), separated, divorced, widowed, and unmarried or domestic partnership (same sex, opposite sex, or unregistered). Patients with an unknown marital status were excluded from the analysis. Participants were divided into married and unmarried groups (including single, separated, divorced, widowed, and unmarried patients or domestic partners).

Patient demographic characteristics included age, race, sex, insurance status, residence, poverty level, and educational level. Race was included because it is an important confounding factor, which may affect stage at diagnosis, treatment, and CSS. Ethnicity was not an exposure variable in this study. Using the self-reported race/ethnicity categories in the US Census, race in the SEER database was categorized as White, Black, and other (ie, American Indian or Alaska Native and Asian or Pacific Islander). Insurance status was categorized as non-Medicaid insured, uninsured, and Medicaid insured. Residence was categorized as metropolitan and nonmetropolitan. Poverty level (percentage of county population living at <200% of the federal poverty level) and educational level (percentage of county population with less than a high school education) were obtained from linked county-level data.^18^ To investigate sex differences in the association of marriage with CSS, participants were further grouped by marital status and sex in cancers that were distributed in both sexes (including lung, kidney, bladder, prostate, colorectal, pancreatic, and breast cancers). Clinical characteristics, such as cancer stage and grade, and receipt of surgery, radiation therapy (RT), and chemotherapy were analyzed. Stage at diagnosis was based on the American Joint Committee on Cancer Staging Atlas, sixth edition^19^; stages I and II were considered early, and stages III and IV were considered advanced. Surgery was defined as undergoing cancer-directed surgery using the SEER surgical codes.^20^ RT was defined as undergoing 1 or more of the following treatments: beam radiation, radioactive implants, radioactive isotopes, and radiation or not specified. Definitive treatment was defined as surgery and/or radiation for prostate, lung, bladder, kidney, pancreatic, and endometrial cancer. Only surgery was considered definitive treatment for breast cancer, colorectal cancer, and melanoma.^3^ CSS was defined as the time between diagnosis and death resulting from the primary cancer through December 31, 2016.

### Statistical Analysis

All statistical analyses were performed using R version 3.6.3 (R Project for Statistical Computing). For all analyses, the *P* values were 2-sided, and the threshold of *P* < .05 was used to determine statistical significance. Continuous variables were compared using the Kruskal-Wallis rank-sum test. Categorical variables were compared using Pearson χ^2^ test. Kaplan-Meier curves were used to illustrate unadjusted associations between marital status and CSS, and the log-rank test was used for comparisons.

Mediation analysis was performed to examine the mechanisms underlying the association between marital status (exposure variable) and CSS (outcome variable) and the role of intermediate factors, including stage at diagnosis and treatment-related variables, such as receiving surgery, chemotherapy, and RT (mediators). Mediation analysis with survival data was conducted using the R package regmedint, based on the product method proposed by Valeri and Vanderweele,^21^ in which 2 regressions are analyzed. We first regressed the outcome *T* with exposure *A*, mediator *M*, and covariate *C* using parametric multivariate accelerated failure time (AFT) regression models with a Weibull distribution^22^ in Equation 1:

log(*T*) = θ_0_ + θ_1_*a* + θ_2_*m* *+* *θ*_3_*am* + *θ′*_4_*c* + νε,

where ε is a random variable following an extreme value distribution and ν is a scale parameter so that *T* follows a Weibull distribution.^23^ The appropriateness of Weibull distribution was validated in eFigure 1 in the Supplement. In AFT models, the time ratio (TR) describes the factor by which the time to event is associated between 2 groups. A TR of *d* for a variable indicates that the ratio of the expected remaining life of this group to the expected remaining life of the reference group is *d*. We then regressed the mediator on the exposure variable and covariates using multivariable logistic regression models in Equation 2:

log{*P*(*M* = 1|*A,C*)} = β_0_ + β_1_*a* + β′_2_*c*.

Next, the natural direct effect (NDE) size (Equation 3), natural indirect effect (NIE) size (Equation 4), and total effect (TE) size (Equation 5) of marital status on CSS were calculated. The NIE size represents the effect size mediated through the mediator. The proportion mediated (PM) (Equation 6), which reflects the magnitude of the effect size mediated through the particular pathway, was further calculated.^24^ A mediator with a significant mediation effect size and a PM greater than 10% was defined as a key mediator*.*
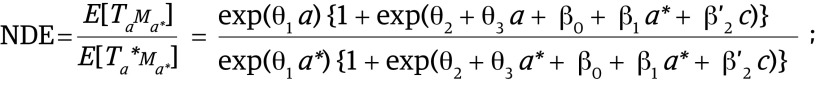


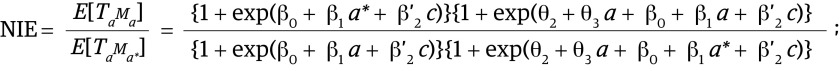

*TE* = *NDE* × *NIE*; and

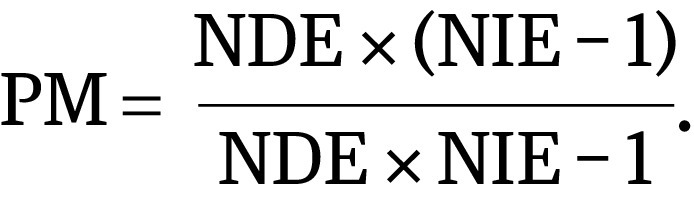
In equations 3 and 4, asterisks indicate that a binary exposure (eg, married) is set to its opposite (ie, unmarried); *E *indicates expected survival time. Patient demographic characteristics (age, race, sex, insurance status, residence, poverty level, and educational level) and grade were included as covariates in the AFT and logistic regression for the mediation analysis of stage at diagnosis. For mediation analysis of treatment-related variables, stage at diagnosis was also adjusted.

## Results

### Patient Demographic and Clinical Characteristics

Data from 1 733 906 patients (894 379 [51.6%] women; mean [SD] age, 63.76 [12.60] years) who were diagnosed with 1 of 9 common cancers from 2007 to 2016 were included in the study. Table 1 presents patient demographic and clinical characteristics, and eTable 1 in the Supplement provides additional information about each cancer. Of all included patients, 1 067 726 (61.6%) were married, and 894 379 (38.4%) were unmarried. The marriage rate was highest in patients with prostate cancer (287 143 of 383 925 [74.8%]) and lowest in those with lung cancer (146 391 of 274 060 [53.4%]). Compared with married patients, unmarried patients were more likely to be older (mean [SD] age, 64.89 [13.79] years vs 63.05 [11.74] years; *P* < .001), to be Black individuals (112 906 [16.9%] vs 88 389 [8.3%]; *P* < .001), to be women (415 698 [62.4%] vs 478 681 [44.78%]; *P* < .001), to lack health insurance (22 719 [3.4%] vs 17 243 [1.6%]; *P* < .001), to live in a metropolitan area (591 974 [88.9%] vs 99 181 [88.0%]; *P* < .001), to live in a county with a higher percentage of the population earning greater than 200% of the federal poverty level (mean [SD] percentage of population, 33.00% [9.50] vs 32.02% [9.57]; *P* < .001), and to live in a county with a higher percentage of the population with a high school educational level (mean [SD] percentage of population, 15.12% [6.10] vs 14.71% [6.20]; *P* < .001). Unmarried patients were more likely to be diagnosed at an advanced stage (237 154 [35.6%] vs 306 795 [28.7%]; *P* < .001) and to undergo chemotherapy (209 702 [31.5%] vs 316 461 [29.6]; *P* < .001) but not surgery (446 642 [67.0%] vs 750 350 [70.3%]; *P* < .001) or RT (217 952 [32.7%] vs 356 513 [33.4%]; *P* < .001). Contrary to the results for the total sample, no difference was found in stage at diagnosis between married and unmarried patients with pancreatic cancer (eTable 1 in the Supplement). Compared with married patients, higher proportions of unmarried patients with prostate, bladder, kidney, and endometrial cancers and melanoma received RT, and lower proportions with breast, lung, colorectal, bladder and pancreatic cancers received chemotherapy (eTable 1 in the Supplement).

**Table 1.** Patient Demographic and Clinical Characteristics by Marital Status

Characteristic	Patients, No. (%)		*P* value
	Married (n = 1 067 726)	Unmarried (n = 666 180)	
Age at diagnosis, mean (SD), y	63.05 (11.74)	64.89 (13.79)	<.001
Race			
White	883 655 (82.8)	509 551 (76.5)	<.001
Black	88 389 (8.3)	112 906 (16.9)	
Other^a^	87 585 (8.2)	39 835 (6.0)	
Unknown	8097 (0.8)	3888 (0.6)	
Sex			
Female	478 681 (44.8)	415 698 (62.4)	<.001
Male	589 045 (55.2)	250 482 (37.6)	
Grade			
I	126 551 (11.9)	80 027 (12.0)	<.001
II	383 913 (36.0)	228 578 (34.3)	
III	309 987 (29.0)	178 684 (26.8)	
IV	35 305 (3.3)	25 483 (3.8)	
Unknown	211 970 (19.9)	153 408 (23.0)	
Surgery			
No	317 376 (29.7)	219 538 (33.0)	<.001
Yes	750 350 (70.3)	446 642 (67.0)	
Radiation			
No or unknown	711 213 (66.6)	448 228 (67.3)	<.001
Yes	356 513 (33.4)	217 952 (32.7)	
Chemotherapy			
No or unknown	751 265 (70.4)	456 478 (68.5)	<.001
Yes	316 461 (29.6)	209 702 (31.5)	
Stage			
Early	760 931 (71.3)	429 026 (64.4)	<.001
Advanced	306 795 (28.7)	237 154 (35.6)	
Insurance			
Insured	960 622 (90.0)	514 788 (77.3)	<.001
Medicaid	64 888 (6.1)	116 159 (17.4)	
Uninsured	17 243 (1.6)	22 719 (3.4)	
Unknown	24 973 (2.3)	12 514 (1.9)	
Poverty, mean (SD), %	32.02 (9.57)	33.00 (9.50)	<.001
Education, mean (SD), %	14.71 (6.20)	15.12 (6.10)	<.001
Residence			
Metropolitan	939 181 (88.0)	591 974 (88.9)	<.001
Nonmetropolitan	127 647 (12.0)	73 611 (11.0)	
Unknown	898 (0.1)	595 (0.1)	

^a^ Includes American Indian or Alaska Native and Asian or Pacific Islander individuals.

### Association of Marital Status With CSS

**Figure 1.**
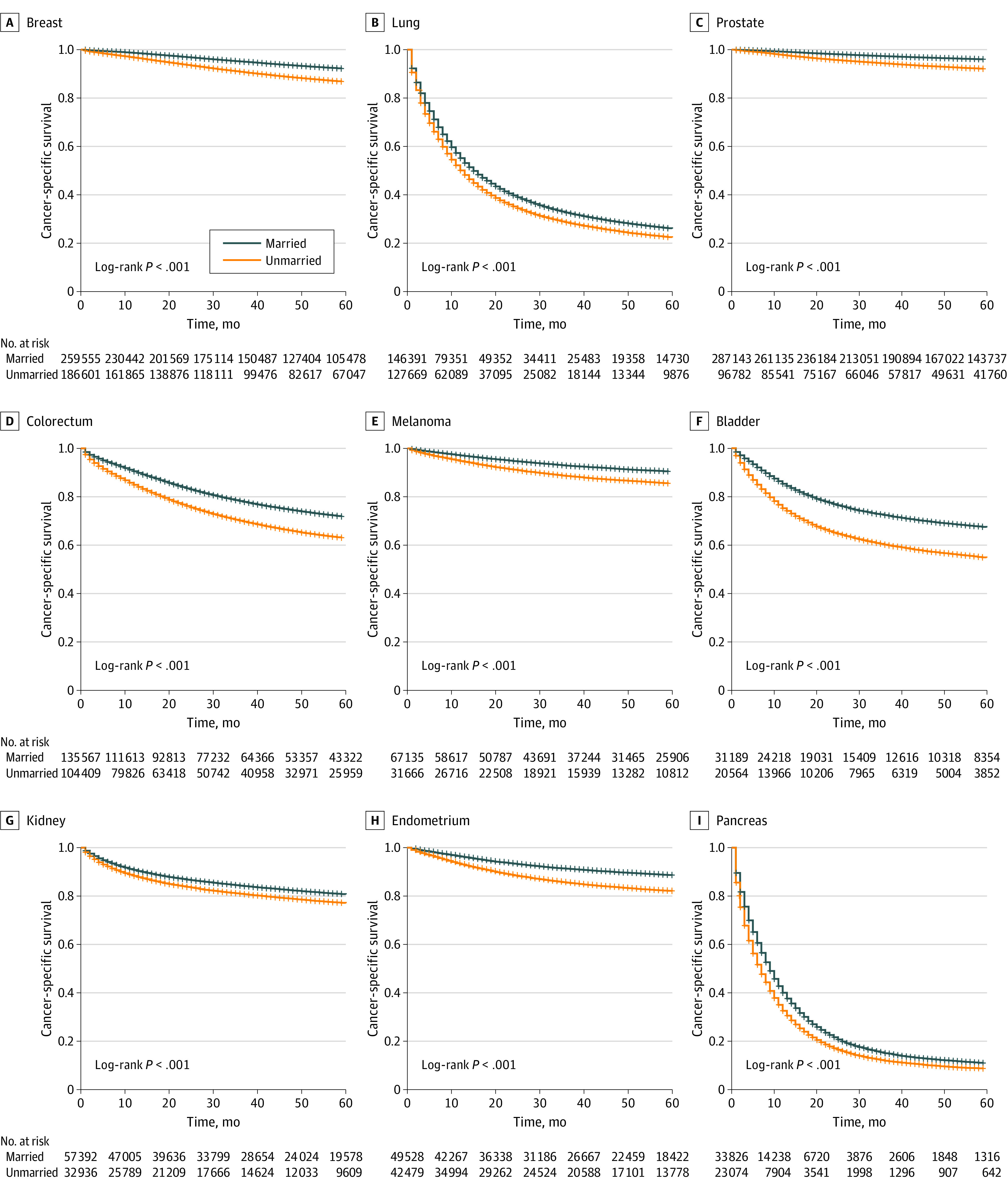
Survival Curves for Cancer-Specific Survival at 1 of 9 Sites

**Figure 2.**
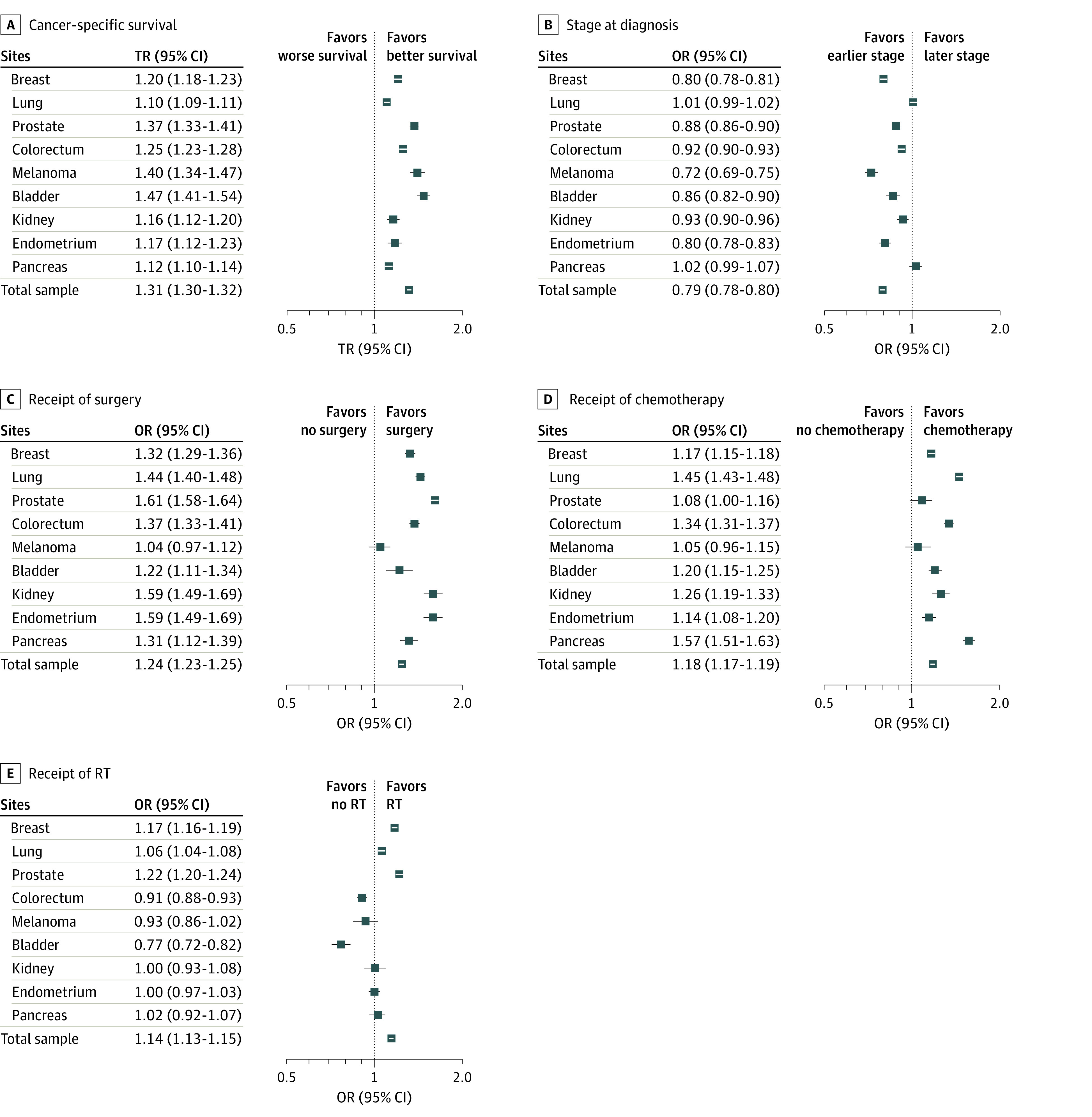
Association Between Marital Status and Cancer-Specific Survival, Diagnosis at an Advanced Stage, and Receipt of Surgery, Chemotherapy, and Radiation Therapy (RT) for Each of the 9 Cancer Sites and the Entire Cohort The time ratio (TR) was adjusted for patient demographic characteristics (age, race, sex, insurance status, residence, poverty level, and educational level), stage, grade, surgery, chemotherapy, and RT. The odds ratio (OR) for diagnosis at an advanced stage was adjusted for patient demographic characteristics and grade. The OR for the surgery was adjusted for patient demographic characteristics, stage, grade, RT, and chemotherapy. The OR for chemotherapy was adjusted for patient demographic characteristics, stage, grade, RT, and surgery. The OR for RT was adjusted for patient demographic characteristics, stage, grade, chemotherapy, and surgery.

Married patients had better 5-year CSS for the cancers analyzed than unmarried patients (Figure 1). After adjusting for patients’ demographic and clinical characteristics, the AFT model showed an association between being married and longer survival (TR, 1.36; 95% CI, 1.35-1.37) (Figure 2A), which remained significant when each cancer was evaluated separately. The TR ranged from 1.10 (95% CI, 1.09-1.11) in lung cancer to 1.47 (95% CI, 1.41-1.54) in bladder cancer (Figure 2A). Married female patients had better 5-year CSS than married male patients for all cancers except bladder cancer (eFigure 2 in the Supplement). Sex disparities in the association of marriage with CSS are shown in Table 2. Improvement in CSS associated with marriage was greater among men than women across all 7 cancer sites (men: TR, 1.27; 95% CI, 1.25-1.28; women: TR, 1.20; 95% CI, 1.19-1.21). Using the AFT regression model, the associations between CSS and stage at diagnosis and undergoing surgery, chemotherapy, and RT were also analyzed for each cancer site (eTable 2 in the Supplement).

**Table 2.** Association of Marital Status With CSS, Advanced-Stage Diagnosis, and Treatment by Cancer Site and Sex

Cancer and sex	CSS		Diagnosed at an advanced stage		Receiving surgery		Receiving chemotherapy		Receiving RT	
	TR for marriage (95% CI)^a^	*P* value	OR for marriage (95% CI)^b^	*P* value	OR for marriage (95% CI)^c^	*P* value	OR for marriage (95% CI)^d^	*P* value	OR for marriage (95% CI)^e^	*P* value
**Lung**										
Male	1.12 (1.11-1.14)	<.001	1.00 (0.98-1.03)	.83	1.53 (1.47-1.59)	<.001	1.06 (1.04-1.09)	<.001	1.57 (1.53-1.61)	<.001
Female	1.08 (1.06-1.10)	<.001	1.02 (1.00-1.05)	.10	1.33 (1.28-1.38)	<.001	1.05 (1.02-1.08)	<.001	1.32 (1.28-1.35)	<.001
**Colorectum**										
Male	1.28 (1.25-1.32)	<.001	0.91 (0.80-0.94)	<.001	1.52 (1.46-1.58)	<.001	0.89 (0.86-0.92)	<.001	1.34 (1.30-1.38)	<.001
Female	1.22 (1.19-1.26)	<.001	0.91 (0.89-0.94)	<.001	1.20 (1.15-1.25)	<.001	0.93 (0.89-0.97)	<.001	1.31 (1.26-1.35)	<.001
**Pancreas**										
Male	1.16 (1.12-1.19)	<.001	1.01 (0.95-1.07)	.77	1.34 (1.22-1.47)	<.001	1.06 (0.99-1.15)	.10	1.63 (1.54-1.72)	<.001
Female	1.08 (1.05-1.11)	<.001	1.06 (1.00-1.11)	.05	1.24 (1.14-1.35)	<.001	0.99 (0.92-1.06)	.71	1.50 (1.42-1.59)	<.001
**Bladder**										
Male	1.50 (1.43-1.57)	<.001	0.81 (0.77-0.85)	<.001	1.25 (1.12-1.39)	<.001	0.76 (0.70-0.82)	<.001	1.17 (1.12-1.23)	<.001
Female	1.40 (1.28-1.53)	<.001	0.96 (0.88-1.04)	.31	1.14 (0.95-1.37)	.15	0.78 (0.68-0.88)	<.001	1.25 (1.15-1.36)	<.001
**Kidney**										
Male	1.18 (1.12-1.23)	<.001	0.91 (0.88-0.95)	<.001	1.76 (1.63-1.91)	<.001	1.03 (0.94-1.14)	.49	1.29 (1.20-1.38)	<.001
Female	1.09 (1.02-1.16)	.009	0.97 (0.92-1.03)	.29	1.31 (1.18-1.45)	<.001	0.95 (0.84-1.08)	.45	1.17 (1.06-1.29)	.001
**Melanoma**										
Male	1.42 (1.34-1.50)	<.001	0.71 (0.67-0.74)	<.001	1.08 (0.99-1.19)	.10	0.94 (0.85-1.05)	.29	1.03 (0.92-1.15)	.66
Female	1.35 (1.24-1.47)	<.001	0.79 (0.74-0.84)	<.001	0.97 (0.86-1.09)	.61	0.91 (0.77-1.06)	.21	1.13 (0.96-1.32)	.14
**Breast**										
Male	1.76 (1.19-2.61)	.005	0.83 (0.69-0.99)	.04	1.05 (0.53-2.03)	.90	1.00 (0.73-1.37)	>.99	1.09 (0.79-1.50)	.59
Female	1.10 (1.07-1.14)	<.001	0.80 (0.78-0.81)	<.001	1.33 (1.26-1.39)	<.001	1.100 (1.07-1.13)	<.001	1.05 (1.03-1.07)	<.001
**Total sample**										
Male	1.27 (1.25-1.28)	<.001	0.85 (0.84-0.86)	<.001	1.43 (1.40-1.46)	<.001	1.33 (1.31-1.35)	<.001	0.91 (0.90-0.93)	<.001
Female	1.20 (1.19-1.21)	<.001	0.83 (0.82-0.84)	<.001	1.31 (1.29-1.33)	<.001	1.23 (1.22-1.25)	<.001	1.16 (1.14-1.17)	<.001

Abbreviations: CSS, cancer-specific survival; OR, odds ratio; RT, radiation therapy; TR, time ratio.

^a^ Adjusted for patient demographic characteristics (age, race, sex, insurance status, residence, poverty level, and educational level), stage, grade, receiving surgery, receiving chemotherapy, and receiving RT.

^b^ Adjusted for patient demographic characteristics and grade.

^c^ Adjusted for patient demographic characteristics, stage, grade, receiving chemotherapy, and receiving RT.

^d^ Adjusted for patient demographic characteristics, stage, grade, receiving surgery, and receiving RT.

^e^ Adjusted for patient demographic characteristics, stage, grade, receiving chemotherapy, and receiving surgery.

### Associations Between Marital Status and Mediators

Multivariate logistic models demonstrated that in the total sample, married patients were more likely than unmarried patients to receive a diagnosis at an early stage (odds ratio [OR], 0.79; 95% CI, 0.78-0.80) and undergo surgery (OR, 1.24; 95% CI, 1.23-1.25), chemotherapy (OR, 1.14; 95% CI, 1.13-1.15), or RT (OR, 1.18; 95% CI, 1.17-1.19) (Figure 2B-2E). However, some data in the single cancer analyses were inconsistent with the total sample. Being married was also associated with not receiving RT for colorectal (OR, 0.906; 95% CI, 0.882-0.931) and bladder (OR, 0.769; 95% CI, 0.721-0.820) cancers (Figure 2E). Our analyses detected no association between marital status and stage at diagnosis in patients with lung and pancreatic cancers, those receiving surgery for melanoma, chemotherapy for melanoma and prostate cancer, or RT for melanoma, kidney, endometrial, and pancreatic cancers (Figure 2B-2E). Given the high heterogeneity between different subtypes of pancreatic cancer, lung cancer, and breast cancer, we further investigated the association between marital status and CSS, advanced stage diagnosis, and treatment for subtypes of these cancers (eTable 3 in the Supplement). Sex disparities in the association between marriage and the mediators and CSS are shown in Table 2. Generally, the associations between marriage and receiving surgery or chemotherapy were greater among men than women. Notably, the associations between marriage and stage at diagnosis and receiving surgery for bladder cancer were significant among men but not women. In addition, the associations between marriage and the stage at diagnosis and treatment for breast cancer were significant among women but not among men.

### Mediation Analyses of Indirect and Direct Effects Sizes for Marriage and CSS

**Figure 3.**
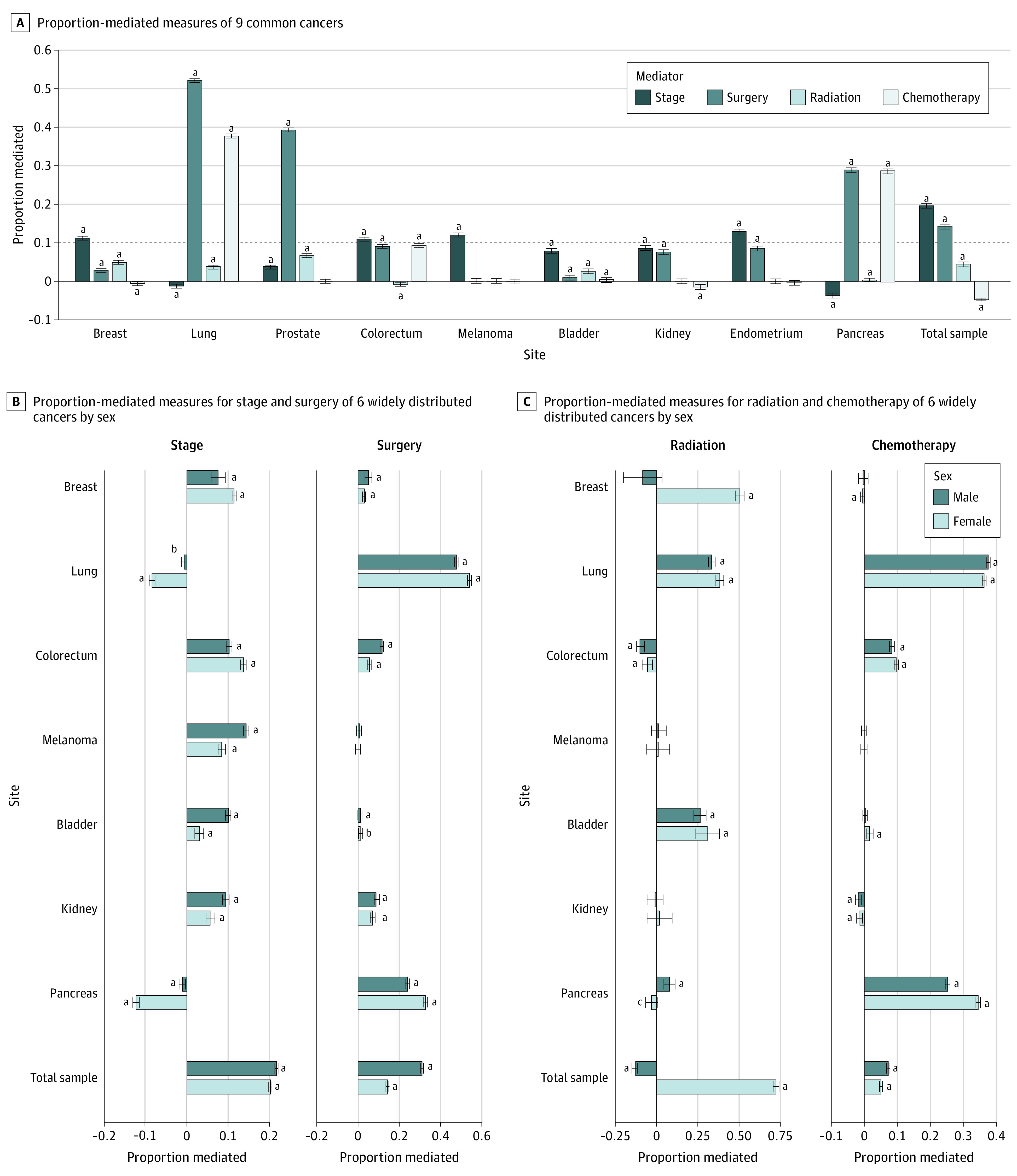
Direct and Indirect Effect Sizes for Marital Status and Cancer-Specific Survival, Mediated by Stage at Diagnosis and Surgery, Chemotherapy, and Radiation Therapy ^a^*P* < .001. ^b^*P* < .01. ^c^*P* < .05.

The NDE, NIE, TE, and PM of marriage on CSS were calculated using mediation analysis and are reported in eTable 4 in the Supplement. The PM for each cancer site is reported in Figure 3. Stage at diagnosis was a key mediator in the association between marriage and CSS in the following cancers: breast (PM, 11.4%; 95% CI, 11.2%-11.6%), colorectal (PM, 10.9%; 95% CI, 10.7%-11.2%), and endometrial (PM, 12.9%; 95% CI, 12.5%-13.3%) cancers and melanoma (PM, 12.0%; 95% CI, 11.7%-12.4%). Surgery was a key mediator of the association of marriage with CSS in lung (PM, 52.2%; 95% CI, 51.9%-52.4%), pancreatic (PM, 28.9%; 95% CI, 28.6%-29.3%), and prostate (PM, 39.3%; 95% CI, 39.0%-39.6%) cancers. Receiving chemotherapy was also a key mediator in lung (PM, 37.7%; 95% CI, 37.6%-37.9%) and pancreatic (PM, 28.6%; 95% CI, 28.4%-28.9%) cancers. Receiving RT was not a key mediator in any of the cancers analyzed. Given the high heterogeneity between different subtypes of pancreatic cancer, lung cancer, and breast cancer, we further calculated the NDE, NIE, TE, and PM of marriage on CSS for subtypes of these cancers (eTable 5 in the Supplement). Furthermore, the subgroup analyses showed that the contributions of stage at diagnosis and treatment with surgery or chemotherapy to the association between marriage and CSS were larger for men than for women (stage at diagnosis: PM, 21.7% [95% CI, 21.5%-21.9%] vs PM, 20.3% [95% CI, 20.2%-20.4%]; surgery: PM, 26.6% [95% CI, 26.4%-26.7%] vs PM, 11.1% [95% CI, 11.0%-11.2%]; chemotherapy: PM, 6.8% [95% CI, 6.7%-6.8%] vs PM, 5.1% [95% CI, 5.0%-5.2%]) (Figure 3B and 3C; eTable 6 in the Supplement). For patients with melanoma, stage at diagnosis was a key mediator for men (PM, 14.4%; 95% CI, 13.9%-14.9%) but not women (PM, 8.4%; 95% CI, 7.7%-9.1%). On the contrary, stage at diagnosis was a key mediator for breast cancer among women (PM, 11.5%; 95% CI, 11.3%-11.7%) but not men (PM, 7.6%; 95% CI, 6.1%-9.0%). Receiving surgery was a key mediator for colorectal cancer among men (PM, 11.7%; 95% CI, 11.5%-11.9%) but not women (PM, 5.4%; 95% CI, 5.1%-5.6%). The analysis of each cancer found that RT was not a key mediator for either sex.

## Discussion

This study evaluated the association between marital status and CSS of patients with 1 of 9 common solid cancers. The extent to which stage at diagnosis and treatment-related factors mediated the survival benefit of marriage was quantified for each of the 9 cancer groups. Sex disparities in the marriage-associated survival benefit and its pathways were examined among the cancers distributed widely in both sexes. To our knowledge, this is the first study to quantitatively identify key mediators of marriage-associated survival benefit, suggesting potential ways of improving CSS in unmarried populations.

Our study found that married patients had a lower risk of presenting with advanced stage disease than unmarried patients for 7 cancers: breast, prostate, colorectal, bladder, kidney, and endometrial cancers and melanoma, which is consistent with previous findings.^3,10,25,26,27^ Early diagnosis was a key mediator in the association between being married and better CSS in only 4 of these 7 cancers (breast, colorectal, and endometrial cancer and melanoma). Several explanations have been proposed for why being married results in earlier diagnoses. Spouses of married individuals promote more positive health-related behaviors, including seeking medical attention for alarming symptoms, receiving cancer screenings, and visiting a doctor regularly.^7^ Published data indicate increased utilization of colorectal, prostate, and breast cancer screenings by married individuals.^28,29,30^ Based on our findings, specific interventions for unmarried individuals should be developed to facilitate their access to primary care and increase their screening rates for breast, colorectal, and endometrial cancers, which may render earlier diagnoses and reduce CSS disparities between married and unmarried populations.

Multivariate logistic regression showed an association between being married and receiving some type of treatment in the cancers analyzed, except melanoma. Consistent with previous findings, we found unmarried patients were less likely to receive definitive treatment than married patients.^3,7,14,31^ However, treatment-related variables were identified as key mediators in the association between marital status and CSS only in lung, pancreatic, and prostate cancers. A widely accepted explanation for the lower odds of unmarried patients receiving definitive treatment is that they have worse adherence to prescribed treatments than married patients.^3,32^ However, a study using SEER data from 925 127 patients by researchers from Harvard, MD Anderson Cancer Center, and the Mayo Clinic^33^ showed that only 0.52% of unmarried patients with cancer declined physician-recommended surgery and 1.33% declined RT. The refusal rates were 0.24% and 0.69%, respectively, among married patients. This discrepancy in refusal rates between married and unmarried patients was small. Another explanation for the discrepancy is based on the stereotype of unmarried patients’ lack of social support and low tolerance for aggressive treatments; thus, physicians may recommend less aggressive treatment for them.^34^ Therefore, for unmarried patients with lung, pancreatic, and prostate cancers, greater effort is needed to provide social supports and eliminate physicians’ biases in their treatment recommendations.

Our results found that married female patients had better CSS than married male patients in the cancers we analyzed, except bladder cancer. The AFT models showed that the association between marriage and improved CSS was higher among men than among women. This finding is consistent with previous studies.^3,35,36^ The reasons for this finding may be a greater tendency for unmarried men than women to engage in health-threatening behavior,^37,38^ better monitoring of spouses’ health-promoting behavior by wives than by husbands,^37,39^ and more social support and social integration provided by wives to husbands than vice versa.^40^ Moreover, women are affected more than men by low-quality marriages,^35^ which may not have the same survival benefits as high-quality marriages.^41^ The exact reasons should be investigated. Marriage was associated with earlier diagnosis and treatment with surgery for men with bladder cancer but not women, which may account for married men’s better CSS compared with married women.

### Limitations

There are limitations in our study. First, patient-level income and education were not available in the SEER database; therefore, our study relied on county-level data for these variables. Second, some important variables related to marriage were not recorded in the SEER database, such as marriage quality, length of marriage, and spouse’s age and income; hence, our study could not include them as covariates. Third, our study did not provide specific information for single, widowed, separated, or divorced populations. Fourth, the demographic and clinical characteristics of excluded individuals were different from those of included individuals (eTable 7 in the Supplement).

## Conclusions

This cohort study found that unmarried patients with 1 of 9 common cancers were at higher risk of cancer mortality compared with married patients. The findings suggest that unmarried patients’ CSS could be improved by promoting early diagnosis of breast, colorectal, and endometrial cancers as well as melanoma and providing definitive treatment for lung, pancreatic, and prostate cancers. The association between marital status and stage at diagnosis for lung and pancreatic cancers needs further investigation. We found that marriage was associated with a more improved CSS in men than in women, and the mediating effect sizes for stage at diagnosis, surgery, and chemotherapy and the association between marital status and CSS was greater for men. The public health system should implement interventions for unmarried individuals to reduce the CSS gap associated with marital status and sex.

## Notes

Supplement. **eFigure 1.** Evaluation of the Appropriateness of Weibull Distribution in AFT Models Assessing the Association Between Marital Status and Cancer-Specific Survival for the Following Cancer Sites: Breast, Lung, Prostate, Colorectum, Melanoma, Bladder, Kidney, Endometrium, and Pancreas **eTable 1.** Patient Demographic and Clinical Characteristics by Marital Status and Cancer Site **eFigure 2.** Survival Curves of Cancer-Specific Survival of Cancer of the Lung, Colorectum, Melanoma, Bladder, Kidney, Pancreas, and Breast by Marital Status and Sex **eTable 2.** Associations Between Cancer-Specific Survival and Marital Status (Married vs Unmarried), Stage at Diagnosis, and Treatment **eTable 3.** Associations Between Cancer-Specific Survival and Marital Status (Married vs Unmarried), Stage at Diagnosis, and Treatment by Cancer Site and Histological Subtype **eTable 4.** Mediation Analyses of Direct and Indirect Effects of Marital Status on Cancer-Specific Survival Mediated by Stage at Diagnosis and Treatment and Receiving Radiation Therapy **eTable 5.** Mediation Analyses of Direct and Indirect Effects of Marital Status on Cancer-Specific Survival Mediated by Stage at Diagnosis and Treatment by Histological Subtype **eTable 6.** Mediation Analyses of Direct and Indirect Effects of Marital Status on Cancer-Specific Survival Mediated by Stage at Diagnosis and Treatment by Sex **eTable 7.** Demographic and Clinical Characteristics of Individuals Included and Excluded
