# Supplementary material for: Assessment of Modifiable Factors for the Association of Marital Status With Cancer-Specific Survival
Source: JAMA Netw Open. 2021 May 28;4(5):e2111813. doi: 10.1001/jamanetworkopen.2021.11813 (PMC8164101; doi:10.1001/jamanetworkopen.2021.11813)
Supplement: Supplement. — eFigure 1. Evaluation of the Appropriateness of Weibull Distribution in AFT Models Assessing the Association Between Marital Status and Cancer-Specific Survival for the Following Cancer Sites: Breast, Lung, Prostate, Colorectum, Melanoma, Bladder, Kidney, Endometrium, and Pancreas eTable 1. Patient Demographic and Clinical Characteristics by Marital Status and Cancer Site eFigure 2. Survival Curves of Cancer-Specific Survival of Cancer of the Lung, Colorectum, Melanoma, Bladder, Kidney, Pancreas, and Breast by Marital Status and Sex eTable 2. Associations Between Cancer-Specific Survival and Marital Status (Married vs Unmarried), Stage at Diagnosis, and Treatment eTable 3. Associations Between Cancer-Specific Survival and Marital Status (Married vs Unmarried), Stage at Diagnosis, and Treatment by Cancer Site and Histological Subtype eTable 4. Mediation Analyses of Direct and Indirect Effects of Marital Status on Cancer-Specific Survival Mediated by Stage at Diagnosis and Treatment and Receiving Radiation Therapy eTable 5. Mediation Analyses of Direct and Indirect Effects of Marital Status on Cancer-Specific Survival Mediated by Stage at Diagnosis and Treatment by Histological Subtype eTable 6. Mediation Analyses of Direct and Indirect Effects of Marital Status on Cancer-Specific Survival Mediated by Stage at Diagnosis and Treatment by Sex eTable 7. Demographic and Clinical Characteristics of Individuals Included and Excluded [file jamanetwopen-e2111813-s001.pdf]

Supplemental Online Content

Chen ZH, Yang KB, Zhang YZ, et al. Assessment of modifiable factors for the association of marital status with cancer-specific survival. *JAMA Netw Open*. 2021;4(5):e2111813. doi:10.1001/jamanetworkopen.2021.11813

eFigure 1. Evaluation of the Appropriateness of Weibull Distribution in AFT Models Assessing the Association Between Marital Status and Cancer-Specific Survival for the Following Cancer Sites: Breast, Lung, Prostate, Colorectum, Melanoma, Bladder, Kidney, Endometrium, and Pancreas
eTable 1. Patient Demographic and Clinical Characteristics by Marital Status and Cancer Site
eFigure 2. Survival Curves of Cancer-Specific Survival of Cancer of the Lung, Colorectum, Melanoma, Bladder, Kidney, Pancreas, and Breast by Marital Status and Sex
eTable 2. Associations Between Cancer-Specific Survival and Marital Status (Married vs Unmarried), Stage at Diagnosis, and Treatment
eTable 3. Associations Between Cancer-Specific Survival and Marital Status (Married vs Unmarried), Stage at Diagnosis, and Treatment by Cancer Site and Histological Subtype
eTable 4. Mediation Analyses of Direct and Indirect Effects of Marital Status on Cancer-Specific Survival Mediated by Stage at Diagnosis and Treatment and Receiving Radiation Therapy
eTable 5. Mediation Analyses of Direct and Indirect Effects of Marital Status on Cancer-Specific Survival Mediated by Stage at Diagnosis and Treatment by Histological Subtype
eTable 6. Mediation Analyses of Direct and Indirect Effects of Marital Status on Cancer-Specific Survival Mediated by Stage at Diagnosis and Treatment by Sex
eTable 7. Demographic and Clinical Characteristics of Individuals Included and Excluded

This supplemental material has been provided by the authors to give readers additional information about their work.

© 2021 Chen ZH et al. *JAMA Network Open.*

eFigure 1. Evaluation of the Appropriateness of Weibull Distribution in AFT Models Assessing the Association Between Marital Status and Cancer-Specific Survival for the Following Cancer Sites: Breast, Lung, Prostate, Colorectum, Melanoma, Bladder, Kidney, Endometrium, and Pancreas

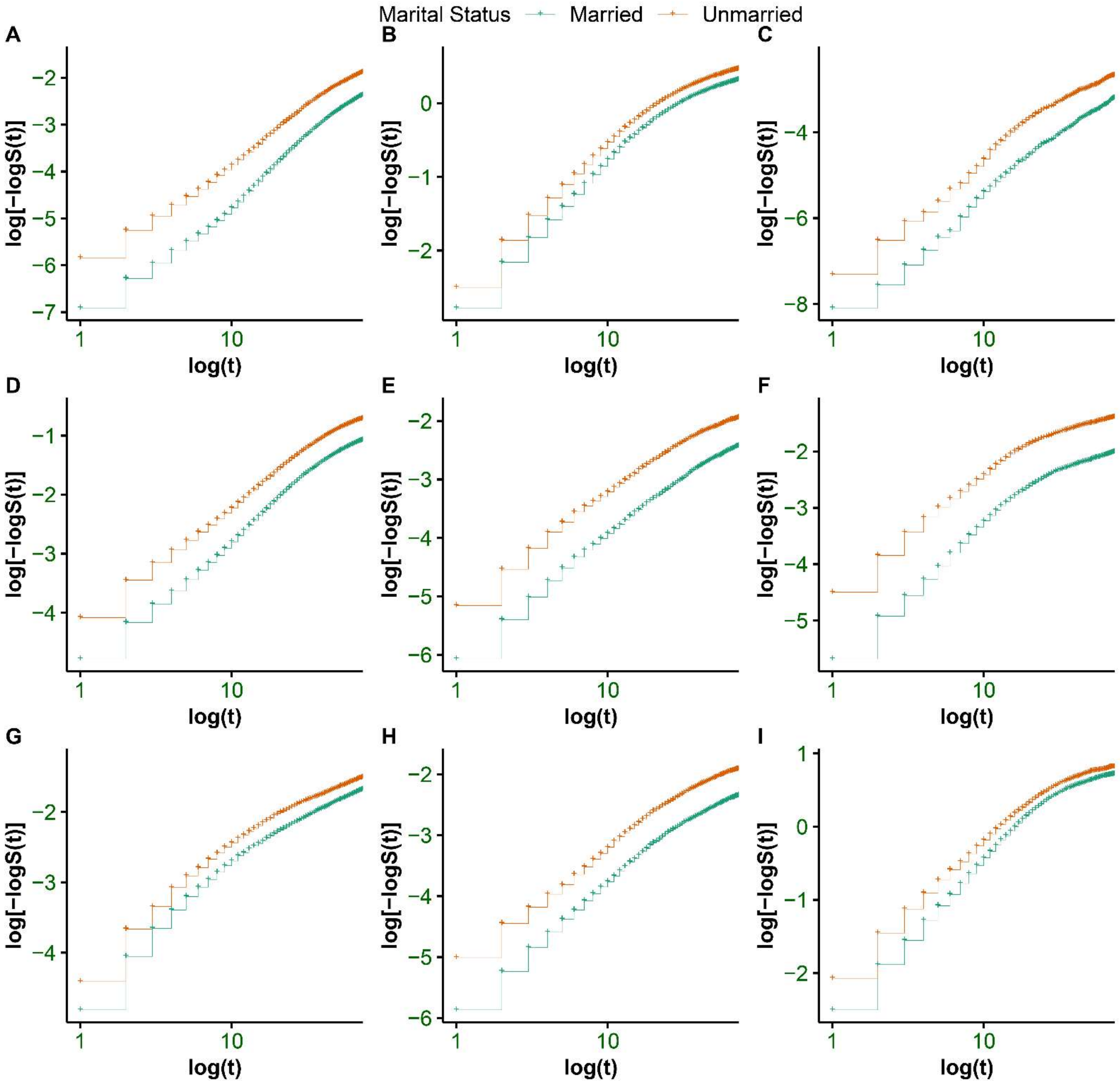

© 2021 Chen ZH et al. *JAMA Network Open.*

eTable 1. Patient Demographic and Clinical Characteristics by Marital Status and Cancer Site

Characteristics	Total Sample			Breast			Lung			Prostate		
	Married	Unmarried	*P*	Married	Unmarried	*P*	Married	Unmarried	*P*	Married	Unmarried	*P*
	**1067726**	**666180**		**259555**	**186601**		**146391**	**127669**		**287143**	**96782**	
**Age at diagnosis**[a]	63.05 (11.74)	64.89 (13.79)	<0.001	58.25 (12.24)	63.16 (14.56)	<0.001	67.26 (10.28)	67.87 (11.43)	<0.001	65.46 (8.71)	65.14 (9.50)	<0.001
**Race**[b]			<0.001			<0.001			<0.001			<0.001
**White**	883655 (82.8)	509551 (76.5)		212617 (81.9)	140483 (75.3)		121240 (82.8)	99304 (77.8)		230879 (80.4)	67121 (69.4)	
**Black**	88389 (8.3)	112906 (16.9)		18009 (6.9)	31463 (16.9)		11267 (7.7)	21225 (16.6)		36093 (12.6)	24908 (25.7)	
**Others**[c]	87585 (8.2)	39835 (6.0)		27613 (10.6)	13714 (7.3)		13626 (9.3)	6917 (5.4)		16621 (5.8)	3580 (3.7)	
**Unknown**	8097 (0.8)	3888 (0.6)		1316 (0.5)	941 (0.5)		258 (0.2)	223 (0.2)		3550 (1.2)	1173 (1.2)	
**Sex**[b]			<0.001			<0.001			<0.001			<0.001
**Female**	478681 (44.8)	415698 (62.4)		257331 (99.1)	185593 (99.5)		56406 (38.5)	73394 (57.5)				
**Male**	589045 (55.2)	250482 (37.6)		2224 (0.9)	1008 (0.5)		89985 (61.5)	54275 (42.5)		287143 (100.0)	96782 (100.0)	
**Grade**[b]			<0.001			<0.001			<0.001			<0.001
**Grade I**	126551 (11.9)	80027 (12.0)		56790 (21.9)	38231 (20.5)		8312 (5.7)	6221 (4.9)		22102 (7.7)	7612 (7.9)	
**Grade II**	383913 (36.0)	228578 (34.3)		108196 (41.7)	76796 (41.2)		26540 (18.1)	21324 (16.7)		119954 (41.8)	37760 (39.0)	
**Grade III**	309987 (29.0)	178684 (26.8)		81178 (31.3)	60111 (32.2)		38553 (26.3)	32959 (25.8)		137400 (47.9)	47277 (48.8)	
**Grade IV**	35305 (3.3)	25483 (3.8)		1192 (0.5)	939 (0.5)		5083 (3.5)	4560 (3.6)		472 (0.2)	240 (0.2)	
**Unknown**	211970 (19.9)	153408 (23.0)		12199 (4.7)	10524 (5.6)		67903 (46.4)	62605 (49.0)		7215 (2.5)	3893 (4.0)	

© 2021 Chen ZH et al. *JAMA Network Open.*

eTable 1 cont.

Characteristics	Total Sample			Breast			Lung			Prostate		
	Married	Unmarried	*P*	Married	Unmarried	*P*	Married	Unmarried	*P*	Married	Unmarried	*P*
	1067726	666180		259555	186601		146391	127669		287143	96782	
**Surgery**[b]			<0.001			<0.001			<0.001			<0.001
**No**	317376 (29.7)	219538 (33.0)		11755 (4.5)	16310 (8.7)		107078 (73.1)	100184 (78.5)		149111 (51.9)	59887 (61.9)	
**Yes**	750350 (70.3)	446642 (67.0)		247800 (95.5)	170291 (91.3)		39313 (26.9)	27485 (21.5)		138032 (48.1)	36895 (38.1)	
**Radiation**[b]			<0.001			<0.001			<0.001			<0.001
**No/Unknown**	711213 (66.6)	448228 (67.3)		117187 (45.1)	96243 (51.6)		79872 (54.6)	71315 (55.9)		187570 (65.3)	61289 (63.3)	
**Yes**	356513 (33.4)	217952 (32.7)		142368 (54.9)	90358 (48.4)		66519 (45.4)	56354 (44.1)		99573 (34.7)	35493 (36.7)	
**Chemotherapy**[b]			<0.001			<0.001			<0.001			<0.001
**No/Unknown**	751265 (70.4)	456478 (68.5)		142721 (55.0)	114570 (61.4)		62616 (42.8)	65693 (51.5)		284576 (99.1)	95550 (98.7)	
**Yes**	316461 (29.6)	209702 (31.5)		116834 (45.0)	72031 (38.6)		83775 (57.2)	61976 (48.5)		2567 (0.9)	1232 (1.3)	
**Stage**[b]			<0.001			<0.001			<0.001			<0.001
**Early**	760931 (71.3)	429026 (64.4)		222976 (85.9)	152277 (81.6)		42196 (28.8)	35925 (28.1)		242680 (84.5)	79180 (81.8)	
**Advanced**	306795 (28.7)	237154 (35.6)		36579 (14.1)	34324 (18.4)		104195 (71.2)	91744 (71.9)		44463 (15.5)	17602 (18.2)	
**Insurance**[b]			<0.001			<0.001			<0.001			<0.001
**Insured**	960622 (90.0)	514788 (77.3)		235173 (90.6)	146260 (78.4)		128801 (88.0)	93827 (73.5)		261955 (91.2)	78997 (81.6)	
**Medicaid**	64888 (6.1)	116159 (17.4)		18208 (7.0)	34021 (18.2)		12855 (8.8)	27039 (21.2)		9962 (3.5)	10932 (11.3)	
**Uninsured**	17243 (1.6)	22719 (3.4)		3280 (1.3)	4151 (2.2)		3134 (2.1)	5139 (4.0)		3031 (1.1)	2844 (2.9)	
**Unknown**	24973 (2.3)	12514 (1.9)		2894 (1.1)	2169 (1.2)		1601 (1.1)	1664 (1.3)		12195 (4.2)	4009 (4.1)	

© 2021 Chen ZH et al. *JAMA Network Open.*

eTable 1 cont.

Characteristics	Total Sample			Breast			Lung			Prostate		
	Married	Unmarried	*P*	Married	Unmarried	*P*	Married	Unmarried	*P*	Married	Unmarried	*P*
	**1067726**	**666180**		**259555**	**186601**		**146391**	**127669**		**287143**	**96782**	
**Poverty**[a]	32.02 (9.57)	33.00 (9.50)	<0.001	31.64 (9.39)	32.70 (9.33)	<0.001	32.97 (9.92)	33.60 (9.77)	<0.001	31.79 (9.53)	33.17 (9.53)	<0.001
**Education**[a]	14.71 (6.20)	15.12 (6.10)	<0.001	14.54 (6.14)	15.01 (6.05)	<0.001	15.08 (6.35)	15.21 (6.20)	<0.001	14.60 (6.13)	15.20 (6.01)	<0.001
**Residence**[b]			<0.001			<0.001			<0.001			<0.001
**Metropolitan**	939181 (88.0)	591974 (88.9)		232835 (89.7)	168658 (90.4)		123404 (84.3)	110042 (86.2)		255010 (88.8)	86575 (89.5)	
**Nonmetropolitan**	127647 (12.0)	73611 (11.0)		26467 (10.2)	17759 (9.5)		22817 (15.6)	17483 (13.7)		32045 (11.2)	10167 (10.5)	
**Unknown**	898 (0.1)	595 (0.1)		253 (0.1)	184 (0.1)		170 (0.1)	144 (0.1)		88 (0.0)	40 (0.0)	

© 2021 Chen ZH et al. *JAMA Network Open.*

eTable 1 cont.

Characteristics	Colorectum			Melanoma			Bladder			Kidney		
	**Married**	**Unmarried**	***P***	**Married**	**Unmarried**	***P***	**Married**	**Unmarried**	***P***	**Married**	**Unmarried**	***P***
	**135567**	**104409**		**67135**	**31666**		**31189**	**20564**		**57392**	**32936**	
**Age at diagnosis[a]**	63.70 (12.82)	66.69 (14.91)	<0.001	59.68 (14.37)	58.03 (19.45)	<0.001	70.30 (11.02)	71.56 (13.10)	<0.001	61.10 (11.89)	60.54 (14.47)	<0.001
**Race[b]**			<0.001			<0.001			<0.001			<0.001
**White**	108889 (80.3)	78895 (75.6)		65035 (96.9)	30506 (96.3)		27826 (89.2)	17461 (84.9)		48239 (84.1)	25338 (76.9)	
**Black**	11268 (8.3)	17295 (16.6)		216 (0.3)	305 (1.0)		1383 (4.4)	2153 (10.5)		4624 (8.1)	5543 (16.8)	
**Others[c]**	14716 (10.9)	7793 (7.5)		609 (0.9)	363 (1.1)		1846 (5.9)	861 (4.2)		4058 (7.1)	1826 (5.5)	
**Unknown**	694 (0.5)	426 (0.4)		1275 (1.9)	492 (1.6)		134 (0.4)	89 (0.4)		471 (0.8)	229 (0.7)	
**Sex[b]**			<0.001			<0.001			<0.001			<0.001
**Female**	53558 (39.5)	60503 (57.9)		26003 (38.7)	16537 (52.2)		4890 (15.7)	7651 (37.2)		17506 (30.5)	15534 (47.2)	
**Male**	82009 (60.5)	43906 (42.1)		41132 (61.3)	15129 (47.8)		26299 (84.3)	12913 (62.8)		39886 (69.5)	17402 (52.8)	
**Grade[b]**			<0.001			<0.001			<0.001			<0.001
**Grade I**	12291 (9.1)	9002 (8.6)		108 (0.2)	37 (0.1)		986 (3.2)	655 (3.2)		5218 (9.1)	3037 (9.2)	
**Grade II**	86670 (63.9)	65058 (62.3)		170 (0.3)	81 (0.3)		2921 (9.4)	1885 (9.2)		22688 (39.5)	12572 (38.2)	
**Grade III**	19407 (14.3)	15809 (15.1)		293 (0.4)	162 (0.5)		6989 (22.4)	4734 (23.0)		13885 (24.2)	7440 (22.6)	
**Grade IV**	3171 (2.3)	2730 (2.6)		103 (0.2)	73 (0.2)		17519 (56.2)	11224 (54.6)		4960 (8.6)	2878 (8.7)	
**Unknown**	14028 (10.3)	11810 (11.3)		66461 (99.0)	31313 (98.9)		2774 (8.9)	2066 (10.0)		10641 (18.5)	7009 (21.3)	

© 2021 Chen ZH et al. *JAMA Network Open.*

eTable 1 cont.

Characteristics	Colorectum			Melanoma			Bladder			Kidney		
	Married	Unmarried	*P*	Married	Unmarried	*P*	Married	Unmarried	*P*	Married	Unmarried	*P*
	135567	104409		67135	31666		31189	20564		57392	32936	
**Surgery[b]**			<0.001			<0.001			<0.001			<0.001
**No**	14010 (10.3)	14956 (14.3)		3035 (4.5)	1905 (6.0)		1125 (3.6)	1132 (5.5)		4986 (8.7)	4141 (12.6)	
**Yes**	121557 (89.7)	89453 (85.7)		64100 (95.5)	29761 (94.0)		30064 (96.4)	19432 (94.5)		52406 (91.3)	28795 (87.4)	
**Radiation[b]**			<0.001			<0.001			<0.001			<0.001
**No/Unknown**	114023 (84.1)	89232 (85.5)		65172 (97.1)	30417 (96.1)		28403 (91.1)	18109 (88.1)		54692 (95.3)	31247 (94.9)	
**Yes**	21544 (15.9)	15177 (14.5)		1963 (2.9)	1249 (3.9)		2786 (8.9)	2455 (11.9)		2700 (4.7)	1689 (5.1)	
**Chemotherapy[b]**			<0.001			<0.001			<0.001			<0.001
**No/Unknown**	73477 (54.2)	62568 (59.9)		65513 (97.6)	30626 (96.7)		20094 (64.4)	13974 (68.0)		51284 (89.4)	29516 (89.6)	
**Yes**	62090 (45.8)	41841 (40.1)		1622 (2.4)	1040 (3.3)		11095 (35.6)	6590 (32.0)		6108 (10.6)	3420 (10.4)	
**Stage[b]**			<0.001			<0.001			<0.001			<0.001
**Early**	74593 (55.0)	55235 (52.9)		59267 (88.3)	26438 (83.5)		24515 (78.6)	15274 (74.3)		40604 (70.7)	22940 (69.7)	
**Advanced**	60974 (45.0)	49174 (47.1)		7868 (11.7)	5228 (16.5)		6674 (21.4)	5290 (25.7)		16788 (29.3)	9996 (30.3)	
**Insurance[b]**			<0.001			<0.001			<0.001			<0.001
**Insured**	119381 (88.1)	78474 (75.2)		60430 (90.0)	25927 (81.9)		28470 (91.3)	16365 (79.6)		51640 (90.0)	24320 (73.8)	
**Medicaid**	10967 (8.1)	19840 (19.0)		1442 (2.1)	2955 (9.3)		1776 (5.7)	3165 (15.4)		3996 (7.0)	6721 (20.4)	
**Uninsured**	3345 (2.5)	4614 (4.4)		965 (1.4)	1152 (3.6)		435 (1.4)	699 (3.4)		1140 (2.0)	1506 (4.6)	
**Unknown**	1874 (1.4)	1481 (1.4)		4298 (6.4)	1632 (5.2)		508 (1.6)	335 (1.6)		616 (1.1)	389 (1.2)	

© 2021 Chen ZH et al. *JAMA Network Open.*

eTable 1 cont.

Characteristics	Colorectum			Melanoma			Bladder			Kidney		
	**Married**	**Unmarried**	***P***	**Married**	**Unmarried**	***P***	**Married**	**Unmarried**	***P***	**Married**	**Unmarried**	***P***
	**135567**	**104409**		**67135**	**31666**		**31189**	**20564**		**57392**	**32936**	
**Poverty[a]**	32.67 (9.64)	33.31 (9.51)	<0.001	30.80 (9.17)	31.47 (9.01)	<0.001	31.98 (9.53)	32.70 (9.52)	<0.001	32.80 (9.69)	33.54 (9.48)	<0.001
**Education[a]**	15.20 (6.33)	15.40 (6.17)	<0.001	13.85 (5.90)	14.38 (5.91)	<0.001	14.72 (6.22)	15.08 (6.16)	<0.001	15.14 (6.38)	15.41 (6.25)	<0.001
**Residence[b]**			<0.001			<0.001			<0.001			<0.001
**Metropolitan**	117408 (86.6)	91984 (88.1)		59427 (88.5)	28367 (89.6)		27222 (87.3)	18088 (88.0)		50057 (87.2)	29148 (88.5)	
**Nonmetropolitan**	17930 (13.2)	12278 (11.8)		7694 (11.5)	3292 (10.4)		3952 (12.7)	2467 (12.0)		7274 (12.7)	3757 (11.4)	
**Unknown**	229 (0.2)	147 (0.1)		14 (0.0)	7 (0.0)		15 (0.0)	9 (0.0)		61 (0.1)	31 (0.1)	

© 2021 Chen ZH et al. *JAMA Network Open.*

eTable 1 cont.

Characteristic	Endometrium			Pancreas		
	Married	Unmarried	*P*	Married	Unmarried	*P*
	**49528**	**42479**		**33826**	**23074**	
**Age at diagnosis[a]**	60.47 (10.40)	62.72 (12.70)	<0.001	65.78 (10.97)	66.91 (12.95)	<0.001
**Race[b]**			<0.001			<0.001
**White**	41197 (83.2)	33210 (78.2)		27733 (82.0)	17233 (74.7)	
**Black**	2774 (5.6)	5836 (13.7)		2755 (8.1)	4178 (18.1)	
**Others[c]**	5235 (10.6)	3169 (7.5)		3261 (9.6)	1612 (7.0)	
**Unknown**	322 (0.7)	264 (0.6)		77 (0.2)	51 (0.2)	
**Sex[b]**			<0.001			<0.001
**Female**	49528 (100.0)	42479 (100.0)		13459 (39.8)	14007 (60.7)	
**Male**				20367 (60.2)	9067 (39.3)	
**Grade[b]**			<0.001			<0.001
**Grade I**	18481 (37.3)	13873 (32.7)		2263 (6.7)	1359 (5.9)	
**Grade II**	10976 (22.2)	9521 (22.4)		5798 (17.1)	3581 (15.5)	
**Grade III**	6877 (13.9)	6891 (16.2)		5405 (16.0)	3301 (14.3)	
**Grade IV**	2436 (4.9)	2621 (6.2)		369 (1.1)	218 (0.9)	
**Unknown**	10758 (21.7)	9573 (22.5)		19991 (59.1)	14615 (63.3)	

© 2021 Chen ZH et al. *JAMA Network Open.*

eTable 1 cont.

Characteristic	Endometrium			Pancreas		
	Married	Unmarried	*P*	Married	Unmarried	*P*
	**49528**	**42479**		**33826**	**23074**	
**Surgery**[b]			<0.001			<0.001
**No**	1729 (3.5)	3012 (7.1)		24547 (72.6)	18011 (78.1)	
**Yes**	47799 (96.5)	39467 (92.9)		9279 (27.4)	5063 (21.9)	
**Radiation**[b]			<0.001			<0.001
**No/Unknown**	37063 (74.8)	31061 (73.1)		27231 (80.5)	19315 (83.7)	
**Yes**	12465 (25.2)	11418 (26.9)		6595 (19.5)	3759 (16.3)	
**Chemotherapy**[b]			<0.001			<0.001
**No/Unknown**	40174 (81.1)	33713 (79.4)		10810 (32.0)	10268 (44.5)	
**Yes**	9354 (18.9)	8766 (20.6)		23016 (68.0)	12806 (55.5)	
**Stage**[b]			<0.001			<0.001
**Early**	40991 (82.8)	32955 (77.6)		13109 (38.8)	8802 (38.1)	
**Advanced**	8537 (17.2)	9524 (22.4)		20717 (61.2)	14272 (61.9)	
**Insurance**[b]			<0.001			<0.001
**Insured**	44571 (90.0)	33131 (78.0)		30201 (89.3)	17487 (75.8)	
**Medicaid**	3161 (6.4)	7142 (16.8)		2521 (7.5)	4344 (18.8)	
**Uninsured**	1194 (2.4)	1684 (4.0)		719 (2.1)	930 (4.0)	
**Unknown**	602 (1.2)	522 (1.2)		385 (1.1)	313 (1.4)	

© 2021 Chen ZH et al. *JAMA Network Open.*

eTable 1 cont.

Characteristic	Endometrium			Pancreas		
	Married	Unmarried	*P*	Married	Unmarried	*P*
	**49528**	**42479**		**33826**	**23074**	
**Poverty**[a]	31.65 (9.52)	32.34 (9.38)	<0.001	32.00 (9.52)	32.75 (9.51)	<0.001
**Education**[a]	14.50 (6.19)	14.88 (6.07)	<0.001	14.72 (6.18)	14.95 (6.07)	<0.001
**Residence**[b]			<0.001			<0.001
**Metropolitan**	43837 (88.5)	38337 (90.2)		29981 (88.6)	20775 (90.0)	
**Nonmetropolitan**	5658 (11.4)	4124 (9.7)		3810 (11.3)	2284 (9.9)	
**Unknown**	33 (0.1)	18 (0.0)		35 (0.1)	15 (0.1)	

[a]mean (SD); [b]N (%); [c]include American Indian, AK Native, Asian and Pacific Islander

© 2021 Chen ZH et al. *JAMA Network Open.*

eFigure 2. Survival Curves of Cancer-Specific Survival of Cancer of the Lung, Colorectum, Melanoma, Bladder, Kidney, Pancreas, and Breast by Marital Status and Sex

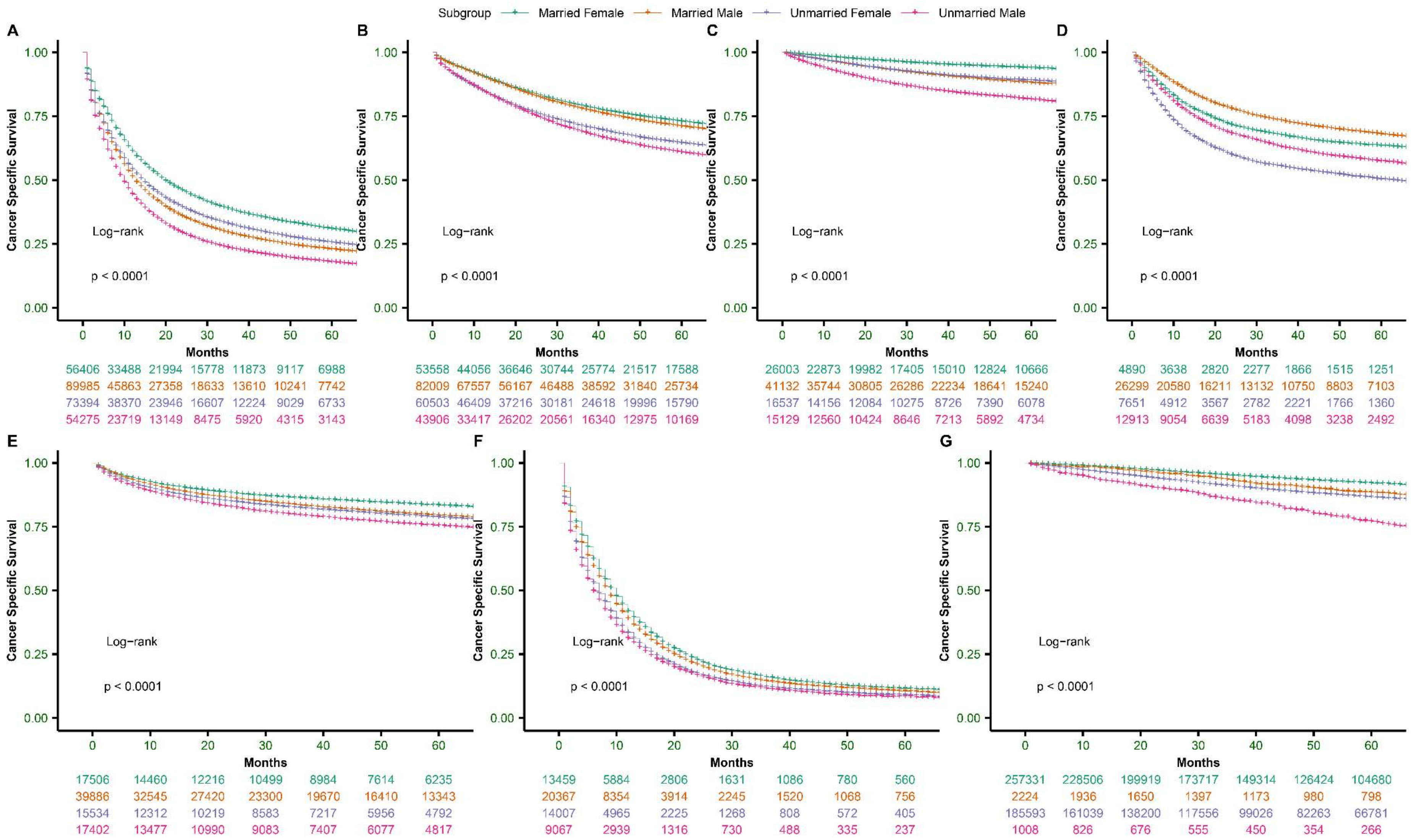

© 2021 Chen ZH et al. *JAMA Network Open.*

eTable 2. Associations Between Cancer-Specific Survival and Marital Status (Married vs Unmarried), Stage at Diagnosis, and Treatment

Risk factors	Total Sample		Breast		Lung		Prostate	
	TR[e] (95%CI)	*P*	TR[e] (95%CI)	*P*	TR[e] (95%CI)	*P*	TR[e] (95%CI)	*P*
**Stage[a]**								
**Early**	Reference		Reference		Reference		Reference	
**Advanced**	0.051 (0.051, 0.052)	<0.001	0.125 (0.122, 0.128)	<0.001	0.158 (0.155, 0.161)	<0.001	0.139 (0.134, 0.144)	<0.001
**Surgery[b]**								
**No**	Reference		Reference		Reference		Reference	
**Yes**	3.442 (3.417, 3.467)	<0.001	3.824 (3.729, 3.922)	<0.001	4.735 (4.638, 4.834)	<0.001	4.137 (3.979, 4.301)	<0.001
**Radiotherapy[c]**								
**No**	Reference		Reference		Reference		Reference	
**Yes**	1.446 (1.436, 1.456)	<0.001	1.251 (1.226, 1.276)	0.015	1.277 (1.263, 1.291)	<0.001	1.587 (1.539, 1.635)	<0.001
**Chemotherapy[d]**								
**No**	Reference		Reference		Reference		Reference	
**Yes**	0.727 (0.722, 0.733)	<0.001	0.973 (0.952, 0.995)	<0.001	1.837 (1.815, 1.86)	<0.001	0.418 (0.396, 0.441)	<0.001

	Colorectum		Melanoma		Bladder		Kidney	
	TR[e] (95%CI)	*P*	TR[e] (95%CI)	*P*	TR[e] (95%CI)	*P*	TR[e] (95%CI)	*P*
**Stage[a]**	Reference		Reference		Reference		Reference	
**Early**	0.100 (0.097, 0.103)	<0.001	0.047 (0.044, 0.05)	<0.001	0.138 (0.131, 0.144)	<0.001	0.042 (0.039, 0.045)	<0.001
**Advanced**								
**Surgery[b]**	Reference		Reference		Reference		Reference	
**No**	6.593 (6.447, 6.743)	<0.001	2.263 (2.128, 2.408)	<0.001	2.335 (2.16, 2.525)	<0.001	6.976 (6.606, 7.367)	<0.001
**Yes**								
**Radiotherapy[c]**	Reference		Reference		Reference		Reference	
**No**	1.25 (1.22, 1.282)	<0.001	0.461 (0.432, 0.492)	<0.001	0.525 (0.497, 0.556)	<0.001	0.591 (0.563, 0.62)	<0.001
**Yes**								
**Chemotherapy[d]**	Reference		Reference		Reference		Reference	
**No**	1.33 (1.302, 1.359)	<0.001	0.485 (0.454, 0.518)	<0.001	1.039 (0.994, 1.086)	0.094	0.662 (0.633, 0.692)	<0.001
**Yes**								

© 2021 Chen ZH et al. *JAMA Network Open.*

eTable 2 cont.

Risk factors	Endometrium		Pancreas	
	TR[e] (95%CI)	*P*	TR[e] (95%CI)	*P*
**Stage[a]**				
**Early**	Reference		Reference	
**Advanced**	0.093 (0.088, 0.099)	<0.001	0.384 (0.375, 0.393)	<0.001
**Surgery[b]**				
**No**	Reference		Reference	
**Yes**	6.582 (6.225, 6.959)	<0.001	3.156 (3.056, 3.259)	<0.001
**Radiotherapy[c]**				
**No**	Reference		Reference	
**Yes**	1.323 (1.266, 1.383)	<0.001	1.152 (1.123, 1.182)	<0.001
**Chemotherapy[d]**				
**No**	Reference		Reference	
**Yes**	0.954 (0.907, 1.003)	0.068	1.548 (1.516, 1.581)	<0.001

[a]After adjusted for age, race, marital status, sex, grade, residence, poverty level and educational level; [b]After adjusted for age, race, marital status, sex, grade, residence, poverty level, educational level, stage at diagnosis, radiotherapy, and chemotherapy; [c]After adjusted for age, race, marital status, sex, grade, residence, poverty level, educational level, stage at diagnosis, surgery, and chemotherapy; [d]After adjusted for age, race, marital status, sex, grade, residence, poverty level, educational level, stage at diagnosis, surgery, and radiotherapy; [e]Time ratio

© 2021 Chen ZH et al. *JAMA Network Open.*

eTable 3. Associations Between Cancer-Specific Survival and Marital Status (Married vs Unmarried), Stage at Diagnosis, and Treatment by Cancer Site and Histological Subtype

	CSS[a]		Diagnosed at an Advanced Stage		Receiving Surgery		Receiving Chemotherapy		Receiving RT[f]	
**Cancer and Sex**	**TR[b] for Marriage (95% CI)**	***P***	**OR[c] for Marriage (95% CI)**	***P***	**OR[d] for Marriage (95% CI)**	***P***	**OR[e] for Marriage (95% CI)**	***P***	**OR[g] for Marriage(95% CI)**	***P***
**Lung**										
**SCLC[h]**	1.072(1.046, 1.099)	<0.001	1.019(0.945, 1.100)	0.621	1.374(1.197, 1.58)	<0.001	1.36(1.284, 1.441)	<0.001	1.127(1.076, 1.179)	<0.001
**NSCLC[i]**	1.105(1.091, 1.118)	<0.001	1.003(0.983, 1.023)	0.774	1.437(1.397, 1.477)	<0.001	1.478(1.45, 1.507)	<0.001	1.046(1.027, 1.066)	<0.001
**Pancreas**										
**Adenocarcinoma**	1.088(1.067, 1.109)	<0.001	1.030(0.990, 1.072)	0.147	1.292(1.212, 1.379)	<0.001	1.615(1.551 ,1.682)	<0.001	1.025(0.975, 1.079)	0.332
**pNET[j]**	1.340(1.154, 1.556)	<0.001	0.884(0.738, 1.058)	0.180	1.253(0.984, 1.596)	0.067	1.078(0.878 ,1.324)	0.472	1.111(0.804, 1.548)	0.529
**Breast**										
**Triple Negative**	1.090(1.023, 1.161)	0.008	0.819(0.762, 0.879)	<0.001	1.255(1.112, 1.416)	<0.001	1.117(1.026 ,1.217)	0.011	1.072(1.01,1 .138)	0.023
**HER2 Enriched**	1.171(1.042, 1.316)	<0.001	0.779(0.708, 0.857)	<0.001	1.369(1.172, 1.599)	<0.001	0.986(0.87, 1.116)	0.821	1.029(0.939, 1.127)	0.544
**Luminal A**	1.110(1.065-1.157)	<0.001	0.835(0.806, 0.865)	<0.001	1.298(1.214, 1.387)	<0.001	1.1(1.069,1. 131)	<0.001	1.052(1.025, 1.078)	<0.001
**Luminal B**	1.093(0.995-1.201)	0.041	0.865(0.808, 0.926)	<0.001	1.447(1.293, 1.62)	<0.001	1.118(1.037 ,1.204)	0.004	1.025(0.967, 1.086)	0.409

[a]Cancer-specific survival; [b]Time ratio, adjusted for patient demographics (age, race, sex, insurance status, residence, poverty level and educational level), stage, grade, receiving surgery, receiving chemotherapy and receiving radiation therapy; [c]Odds ratio, adjusted for patient demographics, and grade; [d]adjusted for patient demographics, stage, grade, receiving chemotherapy and receiving radiation therapy; [e]adjusted for patient demographics, stage, grade, receiving surgery and receiving radiation therapy; [e]adjusted for patient demographics, stage, grade, receiving surgery and receiving radiation therapy; [f]Radiation therapy; [g]adjusted for patient demographics, stage, grade, receiving chemotherapy and receiving surgery; [h]SCLC, small cell lung cancer; [i]NSCLC, non-small cell lung cancer; [j]pNET, pancreatic neuroendocrine tumor.

© 2021 Chen ZH et al. *JAMA Network Open.*

eTable 4. Mediation Analyses of Direct and Indirect Effects of Marital Status on Cancer-Specific Survival Mediated by Stage at Diagnosis and Treatment and Receiving Radiation Therapy

Mediators	Total Sample		Breast		Lung		Prostate	
	Effect size (95%CI)	*P*	Effect size (95%CI)	*P*	Effect size (95%CI)	*P*	Effect size (95%CI)	*P*
**Stage**								
**TE[a]**	0.387 (0.378,0.396)	<0.001	0.275 (0.253,0.296)	<0.001	0.177 (0.163,0.191)	<0.001	0.451 (0.421,0.48)	<0.001
**NIE[b]**	0.065 (0.063,0.067)	<0.001	0.028 (0.026,0.03)	<0.001	-0.002 (-0.01,0.006)	<0.001	0.013 (0.011,0.015)	<0.001
**NDE[c]**	0.322 (0.313,0.331)	<0.001	0.247 (0.226,0.268)	<0.001	0.179 (0.167,0.191)	<0.001	0.437 (0.408,0.467)	<0.001
**PM[d]**	0.196 (0.195,0.197)	<0.001	0.114 (0.112,0.116)	<0.001	-0.013 (-0.015, -0.01)	<0.001	0.036 (0.034,0.038)	<0.001
**Surgery**								
**TE**	0.341 (0.335,0.348)	<0.001	0.191 (0.171,0.21)	<0.001	0.19 (0.177,0.203)	<0.001	0.474 (0.446,0.503)	<0.001
**NIE**	0.034 (0.033,0.036)	<0.001	0.005 (0.004,0.006)	<0.001	0.095 (0.087,0.102)	<0.001	0.161 (0.153,0.168)	<0.001
**NDE**	0.307 (0.301,0.314)	<0.001	0.186 (0.166,0.205)	<0.001	0.096 (0.084,0.107)	<0.001	0.314 (0.286,0.341)	<0.001
**PM**	0.117 (0.116,0.117)	<0.001	0.029 (0.027,0.03)	<0.001	0.522 (0.519,0.524)	<0.001	0.393 (0.39,0.396)	<0.001
**Radiotherapy**								
**TE**	0.318 (0.312,0.325)	<0.001	0.194 (0.175,0.214)	<0.001	0.099 (0.088,0.11)	<0.001	0.333 (0.305,0.361)	<0.001
**NIE**	0.011 (0.01,0.012)	<0.001	0.009 (0.008,0.01)	<0.001	0.004 (0.002,0.005)	<0.001	0.019 (0.017,0.021)	<0.001
**NDE**	0.307 (0.301,0.314)	<0.001	0.186 (0.166,0.205)	<0.001	0.096 (0.084,0.107)	<0.001	0.314 (0.286,0.341)	<0.001
**PM**	0.04 (0.04,0.041)	<0.001	0.05 (0.048,0.051)	<0.001	0.037 (0.036,0.038)	<0.001	0.067 (0.065,0.069)	<0.001
**Chemotherapy**								
**TE**	0.298 (0.292,0.305)	<0.001	0.185 (0.165,0.204)	<0.001	0.149 (0.138,0.161)	<0.001	0.313 (0.286,0.341)	<0.001
**NIE**	-0.009 (-0.009,-0.008)	<0.001	-0.001 (-0.002,0)	0.016	0.054 (0.051,0.057)	<0.001	0 (0,0)	0.051
**NDE**	0.307 (0.301,0.314)	<0.001	0.186 (0.166,0.205)	<0.001	0.096 (0.084,0.107)	<0.001	0.314 (0.286,0.341)	<0.001
**PM**	-0.034 (-0.035,-0.034)	<0.001	-0.006 (-0.008,-0.004)	<0.001	0.377 (0.376,0.379)	<0.001	0 (-0.002,0.002)	0.707

© 2021 Chen ZH et al. *JAMA Network Open.*

eTable 4 cont.

Mediators	Colorectum		Melanoma		Bladder		Kidney	
	Effect size (95%CI)	*P*	Effect size (95%CI)	*P*	Effect size (95%CI)	*P*	Effect size (95%CI)	*P*
**Stage**								
**TE[a]**	0.351 (0.329,0.372)	<0.001	0.386 (0.336,0.437)	<0.001	0.442 (0.398,0.486)	<0.001	0.248 (0.204,0.292)	<0.001
**NIE[b]**	0.033 (0.026,0.04)	<0.001	0.039 (0.034,0.044)	<0.001	0.029 (0.02,0.037)	<0.001	0.019 (0.01,0.027)	<0.001
**NDE[c]**	0.318 (0.298,0.338)	<0.001	0.347 (0.297,0.397)	<0.001	0.413 (0.37,0.456)	<0.001	0.229 (0.186,0.273)	<0.001
**PM[d]**	0.109 (0.107,0.112)	<0.001	0.12 (0.117,0.124)	<0.001	0.079 (0.075,0.082)	<0.001	0.085 (0.081,0.09)	<0.001
**Surgery**								
**TE**	0.244 (0.226,0.263)	<0.001	0.338 (0.29,0.385)	<0.001	0.389 (0.347,0.432)	<0.001	0.159 (0.122,0.197)	<0.001
**NIE**	0.02 (0.018,0.021)	<0.001	0 (0,0.001)	0.269	0.003 (0.002,0.005)	<0.001	0.011 (0.01,0.013)	<0.001
**NDE**	0.225 (0.207,0.243)	<0.001	0.337 (0.29,0.385)	<0.001	0.386 (0.344,0.429)	<0.001	0.148 (0.111,0.185)	<0.001
**PM**	0.09 (0.088,0.091)	<0.001	0.001 (-0.002,0.005)	0.387	0.009 (0.007,0.012)	<0.001	0.076 (0.072,0.079)	<0.001
**Radiotherapy**								
**TE**	0.223 (0.205,0.242)	<0.001	0.338 (0.29,0.385)	<0.001	0.395 (0.353,0.437)	<0.001	0.148 (0.111,0.185)	<0.001
**NIE**	-0.002 (-0.002,-0.001)	0.004	0 (0,0.001)	0.12	0.009 (0.006,0.011)	<0.001	0 (0,0)	<0.001
**NDE**	0.225 (0.207,0.243)	<0.001	0.337 (0.29,0.385)	<0.001	0.386 (0.344,0.429)	<0.001	0.148 (0.111,0.185)	<0.001
**PM**	-0.008 (-0.009,-0.006)	<0.001	0.001 (-0.002,0.004)	0.474	0.026 (0.024,0.029)	<0.001	0 (-0.004,0.003)	<0.001
**Chemotherapy**								
**TE**	0.245 (0.227,0.264)	<0.001	0.337 (0.29,0.385)	<0.001	0.388 (0.345,0.43)	<0.001	0.146 (0.108,0.183)	<0.001
**NIE**	0.021 (0.018,0.023)	<0.001	0 (0,0)	0.309	0.002 (0,0.003)	0.102	-0.002 (-0.003,-0.002)	<0.001
**NDE**	0.225 (0.207,0.243)	<0.001	0.337 (0.29,0.385)	<0.001	0.386 (0.344,0.429)	<0.001	0.148 (0.111,0.185)	<0.001
**PM**	0.094 (0.092,0.095)	<0.001	-0.001 (-0.004,0.003)	0.762	0.005 (0.002,0.008)	0.001	-0.015 (-0.019,-0.012)	<0.001

© 2021 Chen ZH et al. *JAMA Network Open.*

eTable 4 cont.

Mediators	Endometrium		Pancreas	
	Effect size (95%CI)	*P*	Effect size (95%CI)	*P*
**Stage**				
**TE[a]**	0.274 (0.226,0.322)	<0.001	0.166 (0.143,0.189)	<0.001
**NIE[b]**	0.031 (0.026,0.037)	<0.001	-0.006 (-0.015,0.004)	<0.001
**NDE[c]**	0.243 (0.195,0.29)	<0.001	0.172 (0.15,0.193)	<0.001
**PM[d]**	0.129 (0.125,0.133)	<0.001	-0.037 (-0.04,-0.033)	<0.001
**Surgery**				
**TE**	0.173 (0.13,0.217)	<0.001	0.153 (0.131,0.176)	<0.001
**NIE**	0.014 (0.012,0.016)	<0.001	0.042 (0.032,0.052)	<0.001
**NDE**	0.16 (0.116,0.203)	<0.001	0.111 (0.091,0.131)	<0.001
**PM**	0.086 (0.082,0.09)	<0.001	0.289 (0.286,0.293)	<0.001
**Radiotherapy**				
**TE**	0.16 (0.116,0.203)	<0.001	0.112 (0.091,0.132)	<0.001
**NIE**	0 (-0.002,0.002)	<0.001	0 (0,0.001)	0.392
**NDE**	0.16 (0.116,0.203)	<0.001	0.111 (0.091,0.131)	<0.001
**PM**	-0.001 (-0.005,0.003)	<0.001	0.004 (0.002,0.006)	<0.001
**Chemotherapy**				
**TE**	0.159 (0.115,0.203)	<0.001	0.153 (0.132,0.173)	<0.001
**NIE**	-0.001 (-0.001,0)	0.076	0.041 (0.037,0.045)	<0.001
**NDE**	0.16 (0.116,0.203)	<0.001	0.111 (0.091,0.131)	<0.001
**PM**	-0.004 (-0.008,0)	0.059	0.286 (0.284,0.289)	<0.001

[a]Total effect; [b]Natural indirect effect; [c]Natural direct effect; [d]Proportion mediated

© 2021 Chen ZH et al. *JAMA Network Open.*

eTable 5. Mediation Analyses of Direct and Indirect Effects of Marital Status on Cancer-Specific Survival Mediated by Stage at Diagnosis and Treatment by Histological Subtype

Mediators	Lung				Pancreas			
	NSCLC[e]		SCLC[f]		Adenocarcinoma		pNET[g]	
	Effect size (95%CI)	*P*	Effect size (95%CI)	*P*	Effect size (95%CI)	*P*	Effect size (95%CI)	*P*
**Stage**								
TE[a]	0.191 (0.175,0.207)	<0.001	0.107 (0.078,0.135)	<0.001	0.156 (0.133,0.178)	<0.001	0.359 (0.189,0.53)	<0.001
NIE[b]	-0.001 (-0.01,0.007)	0.774	-0.003 (-0.014,0.009)	0.621	-0.007 (-0.016,0.002)	0.147	0.052 (-0.024,0.127)	0.183
NDE[c]	0.193 (0.179,0.206)	<0.001	0.11 (0.083,0.136)	<0.001	0.163 (0.142,0.183)	<0.001	0.308 (0.155,0.461)	<0.001
PM[d]	-0.007 (-0.01,-0.004)	<0.001	-0.029 (-0.033,-0.024)	<0.001	-0.048 (-0.051,-0.044)	<0.001	0.166 (0.14,0.192)	<0.001
**Surgery**								
TE	0.208 (0.193,0.222)	<0.001	0.077 (0.052,0.102)	<0.001	0.118 (0.096,0.139)	<0.001	0.351 (0.189,0.514)	<0.001
NIE	0.108 (0.1,0.116)	<0.001	0.007 (0.002,0.012)	0.004	0.034 (0.025,0.042)	<0.001	0.059 (-0.005,0.122)	0.073
NDE	0.1 (0.087,0.112)	<0.001	0.07 (0.045,0.094)	<0.001	0.084 (0.064,0.103)	<0.001	0.293 (0.143,0.442)	<0.001
PM	0.546 (0.543,0.549)	<0.001	0.096 (0.093,0.099)	<0.001	0.299 (0.296,0.302)	<0.001	0.192 (0.169,0.214)	<0.001
**Radiotherapy**								
TE	0.102 (0.089,0.114)	<0.001	0.086 (0.06,0.111)	<0.001	0.084 (0.065,0.104)	<0.001	0.292 (0.143,0.441)	<0.001
NIE	0.002 (0.001,0.003)	<0.001	0.016 (0.01,0.022)	<0.001	0.001 (-0.001,0.002)	0.330	-0.001 (-0.003,0.002)	0.666
NDE	0.1 (0.087,0.112)	<0.001	0.07 (0.045,0.094)	<0.001	0.084 (0.064,0.103)	<0.001	0.293 (0.143,0.442)	<0.001
PM	0.02 (0.019,0.022)	<0.001	0.19 (0.187,0.193)	<0.001	0.007 (0.005,0.009)	0.036	-0.002 (-0.014,0.009))	0.669
**Chemotherapy**								
TE	0.159 (0.146,0.171)	<0.001	0.094 (0.069,0.119)	<0.001	0.139 (0.119,0.159)	<0.001	0.291 (0.141,0.44)	<0.001
NIE	0.059 (0.056,0.062)	<0.001	0.024 (0.019,0.029)	<0.001	0.055 (0.05,0.06)	<0.001	-0.002 (-0.007,0.003)	0.496
NDE	0.1 (0.087,0.112)	<0.001	0.07 (0.045,0.094)	<0.001	0.084 (0.064,0.103)	<0.001	0.293 (0.143,0.442)	<0.001
PM	0.391 (0.389,0.392)	<0.001	0.265 (0.262,0.268)	<0.001	0.411 (0.409,0.413)	<0.001	-0.007 (-0.018,0.004)	0.219

[a]Total effect; [b]Natural indirect effect; [c]Natural direct effect; [d]Proportion mediated; [e]NSCLC, non-small cell lung cancer; [f]SCLC, small cell lung cancer; [g]pNET, pancreatic neuroendocrine tumor.

© 2021 Chen ZH et al. *JAMA Network Open.*

eTable 5 cont.

Mediators	Breast							
	Triple Negative		HER2 Enriched		Luminal A		Luminal B	
	Effect size (95%CI)	*P*	Effect size (95%CI)	*P*	Effect size (95%CI)	*P*	Effect size (95%CI)	*P*
**Stage**								
**TE**[a]	0.163 (0.094,0.231)	<0.001	0.272 (0.145,0.399)	0.002	0.181 (0.136,0.225)	<0.001	0.232 (0.132,0.333)	<0.001
**NIE**[b]	0.032 (0.02,0.044)	<0.001	0.054 (0.032,0.075)	<0.001	0.018 (0.014,0.022)	<0.001	0.025 (0.013,0.036)	<0.001
**NDE**[c]	0.131 (0.063,0.198)	<0.001	0.219 (0.094,0.344)	0.008	0.163 (0.118,0.207)	<0.001	0.208 (0.108,0.307)	<0.001
**PM**[d]	0.21 (0.202,0.217)	<0.001	0.219 (0.207,0.231)	<0.001	0.108 (0.103,0.112)	<0.001	0.117 (0.108,0.126)	<0.001
**Surgery**								
**TE**	0.091 (0.028,0.155)	<0.001	0.169 (0.052,0.286)	0.004	0.107 (0.066,0.148)	<0.001	0.098 (0.004,0.193)	0.040
**NIE**	0.005 (0.002,0.008)	<0.001	0.012 (0.003,0.02)	0.007	0.003 (0.002,0.003)	<0.001	0.009 (0.006,0.012)	<0.001
**NDE**	0.086 (0.022,0.149)	0.007	0.158 (0.041,0.274)	0.008	0.104 (0.063,0.146)	<0.001	0.089 (-0.005,0.183)	0.063
**PM**	0.06 (0.053,0.066)	<0.001	0.074 (0.063,0.085)	<0.001	0.026 (0.022,0.03)	<0.001	0.1 (0.09,0.109)	0.136
**Radiotherapy**								
**TE**	0.089 (0.025,0.152)	0.006	0.157 (0.04,0.274)	0.008	0.106 (0.064,0.147)	<0.001	0.09 (-0.004,0.184)	0.061
**NIE**	0.003 (0,0.006)	0.039	-0.001 (-0.002,0.001)	0.592	0.001 (0,0.002)	0.003	0.001 (-0.001,0.002)	0.437
**NDE**	0.086 (0.022,0.149)	<0.001	0.158 (0.041,0.274)	0.008	0.104 (0.063,0.146)	<0.001	0.089 (-0.005,0.183)	0.063
**PM**	0.033 (0.027,0.04)	0.008	-0.003 (-0.014,0.007)	0.529	0.012 (0.007,0.016)	0.745	0.008 (-0.002,0.018)	0.101
**Chemotherapy**								
**TE**	0.087 (0.023,0.15)	0.007	0.157 (0.04,0.274)	0.009	0.103 (0.061,0.144)	<0.001	0.096 (0.001,0.19)	0.047
**NIE**	0.001 (0,0.002)	0.177	-0.001 (-0.006,0.005)	0.822	-0.002 (-0.003,-0.001)	0.002	0.007 (0.002,0.011)	0.005
**NDE**	0.086 (0.022,0.149)	0.008	0.158 (0.041,0.274)	0.008	0.104 (0.063,0.146)	<0.001	0.089 (-0.005,0.183)	0.063
**PM**	0.01 (0.003,0.016)	0.003	-0.004 (-0.015,0.007)	0.449	-0.019 (-0.023,-0.015)	<0.001	0.072 (0.062,0.081)	<0.001

© 2021 Chen ZH et al. *JAMA Network Open.*

eTable 6. Mediation Analyses of Direct and Indirect Effects of Marital Status on Cancer-Specific Survival Mediated by Stage at Diagnosis and Treatment by Sex

Mediators	Total Sample				Lung				Colorectum			
	Female		Male		Female		Male		Female		Male	
	Effect size (95%CI)	*P*	Effect size (95%CI)	*P*	Effect size (95%CI)	*P*	Effect size (95%CI)	*P*	Effect size (95%CI)	*P*	Effect size (95%CI)	*P*
**Stage**												
**TE**[a]	0.261 (0.248,0.274)	<0.001	0.372 (0.358,0.386)	<0.001	0.129 (0.107,0.15)	<0.001	0.21 (0.19,0.229)	<0.001	0.261 (0.231,0.291)	<0.001	0.405 (0.377,0.434)	<0.001
**NIE**[b]	0.048 (0.045,0.051)	<0.001	0.07 (0.064,0.075)	<0.001	-0.01 (-0.022,0.002)	0.001	-0.001 (-0.012,0.01)	<0.001	0.061 (0.05,0.072)	<0.001	0.035 (0.025,0.044)	<0.001
**NDE**[c]	0.213 (0.201,0.226)	<0.001	0.302 (0.29,0.315)	<0.001	0.139 (0.121,0.157)	<0.001	0.211 (0.195,0.227)	<0.001	0.2 (0.172,0.228)	<0.001	0.371 (0.343,0.398)	<0.001
**PM**[d]	0.203 (0.202,0.204)	<0.001	0.217 (0.215,0.219)	<0.001	-0.084 (-0.089, -0.08)	<0.001	-0.006 (-0.01,-0.002)	<0.001	0.257 (0.253,0.261)	<0.001	0.103 (0.099,0.106)	<0.001
**Surgery**												
**TE**	0.203 (0.194,0.213)	<0.001	0.308 (0.297,0.318)	<0.001	0.161 (0.141,0.182)	<0.001	0.211 (0.194,0.229)	<0.001	0.21 (0.182,0.239)	<0.001	0.278 (0.254,0.303)	<0.001
**NIE**	0.021 (0.019,0.022)	<0.001	0.073 (0.069,0.077)	<0.001	0.085 (0.073,0.097)	<0.001	0.096 (0.088,0.105)	<0.001	0.01 (0.008,0.013)	<0.001	0.029 (0.026,0.032)	<0.001
**NDE**	0.183 (0.173,0.192)	<0.001	0.235 (0.225,0.244)	<0.001	0.076 (0.059,0.093)	<0.001	0.115 (0.1,0.13)	<0.001	0.2 (0.172,0.228)	<0.001	0.249 (0.225,0.274)	<0.001
**PM**	0.111 (0.11,0.112)	<0.001	0.266 (0.264,0.267)	<0.001	0.547 (0.544,0.551)	<0.001	0.483 (0.48,0.485)	<0.001	0.054 (0.051,0.056)	<0.001	0.117 (0.115,0.119)	<0.001

© 2021 Chen ZH et al. *JAMA Network Open.*

eTable 6 cont.

Mediators	Total Sample				Lung				Colorectum			
	Female		Male		Female		Male		Female		Male	
	Effect size (95%CI)	*P*	Effect size (95%CI)	*P*	Effect size (95%CI)	*P*	Effect size (95%CI)	*P*	Effect size (95%CI)	*P*	Effect size (95%CI)	*P*
**Radiotherapy**												
**TE**	0.193 (0.184,0.203)	<0.001	0.233 (0.223,0.242)	<0.001	0.079 (0.062,0.096)	<0.001	0.119 (0.103,0.134)	<0.001	0.199 (0.171,0.227)	<0.001	0.247 (0.223,0.272)	<0.001
**NIE**	0.01 (0.01,0.011)	<0.001	-0.002 (-0.003,-0.002)	<0.001	0.003 (0.001,0.004)	<0.001	0.004 (0.002,0.005)	<0.001	-0.001 (-0.002,0)	<0.001	-0.002 (-0.003,-0.001)	<0.001
**NDE**	0.183 (0.173,0.192)	<0.001	0.235 (0.225,0.244)	<0.001	0.076 (0.059,0.093)	<0.001	0.115 (0.1,0.13)	<0.001	0.2 (0.172,0.228)	<0.001	0.249 (0.225,0.274)	<0.001
**PM**	0.059 (0.058,0.06)	<0.001	-0.01 (-0.011,-0.009)	<0.001	0.038 (0.037,0.04)	<0.001	0.033 (0.032,0.035)	<0.001	-0.006 (-0.008,-0.003)	<0.001	-0.01 (-0.012,-0.008)	<0.001
**Chemotherapy**												
**TE**	0.192 (0.182,0.201)	<0.001	0.25 (0.24,0.26)	<0.001	0.117 (0.1,0.134)	<0.001	0.178 (0.163,0.194)	<0.001	0.219 (0.191,0.248)	<0.001	0.269 (0.245,0.294)	<0.001
**NIE**	0.009 (0.008,0.01)	<0.001	0.015 (0.014,0.016)	<0.001	0.041 (0.037,0.045)	<0.001	0.063 (0.059,0.067)	<0.001	0.019 (0.016,0.022)	<0.001	0.02 (0.017,0.023)	<0.001
**NDE**	0.183 (0.173,0.192)	<0.001	0.235 (0.225,0.244)	<0.001	0.076 (0.059,0.093)	<0.001	0.115 (0.1,0.13)	<0.001	0.2 (0.172,0.228)	<0.001	0.249 (0.225,0.274)	<0.001
**PM**	0.051 (0.05,0.052)	<0.001	0.068 (0.067,0.068)	<0.001	0.363 (0.361,0.365)	<0.001	0.375 (0.373,0.377)	<0.001	0.096 (0.093,0.099)	<0.001	0.083 (0.081,0.086)	<0.001

© 2021 Chen ZH et al. *JAMA Network Open.*

eTable 6 cont.

Mediators	Melanoma				Bladder				Kidney			
	Female		Male		Female		Male		Female		Male	
	Effect size (95%CI)	*P*	Effect size (95%CI)	*P*	Effect size (95%CI)	*P*	Effect size (95%CI)	*P*	Effect size (95%CI)	*P*	Effect size (95%CI)	*P*
**Stage**												
**TE[a]**	0.313 (0.224,0.402)	<0.001	0.409 (0.347,0.471)	<0.001	0.373 (0.281,0.464)	<0.001	0.469 (0.419,0.518)	<0.001	0.117 (0.042,0.192)	<0.001	0.294 (0.24,0.349)	<0.001
**NIE[b]**	0.023 (0.017,0.029)	<0.001	0.05 (0.042,0.057)	<0.001	0.009 (-0.009,0.028)	0.281	0.038 (0.029,0.048)	<0.001	0.006 (- 0.005,0.018)	<0.001	0.025 (0.013,0.036)	0.115
**NDE[c]**	0.29 (0.201,0.379)	<0.001	0.359 (0.298,0.421)	<0.001	0.363 (0.274,0.453)	<0.001	0.431 (0.382,0.479)		0.111 (0.036,0.185)	<0.001	0.27 (0.216,0.323)	<0.001
**PM[d]**	0.084 (0.077,0.091)	<0.001	0.144 (0.139,0.149)	<0.001	0.03 (0.022,0.039)	<0.001	0.1 (0.096,0.105)	<0.001	0.057 (0.049,0.065)	<0.001	0.095 (0.09,0.101)	<0.001
**Surgery**												
**TE**	0.299 (0.215,0.383)	<0.001	0.35 (0.292,0.408)	<0.001	0.341 (0.253,0.429)	<0.001	0.405 (0.358,0.453)	<0.001	0.173 (0.13,0.217)	<0.001	0.177 (0.13,0.224)	<0.001
**NIE**	0 (-0.001,0.001)	0.604	0.001 (0,0.002)	0.103	0.003 (-0.001,0.006)	0.147	0.003 (0.001,0.005)	<0.001	0.014 (0.012,0.016)	<0.001	0.014 (0.012,0.017)	<0.001
**NDE**	0.299 (0.215,0.383)	<0.001	0.349 (0.291,0.407)	<0.001	0.338 (0.25,0.426)	<0.001	0.402 (0.354,0.45)	<0.001	0.16 (0.116,0.203)	<0.001	0.162 (0.116,0.209)	<0.001
**PM**	-0.001 (-0.007,0.005)	0.736	0.003 (-0.001,0.007)	0.136	0.009 (0.003,0.016)	0.003	0.009 (0.006,0.012)	<0.001	0.086 (0.082,0.09)	<0.001	0.088 (0.084,0.093)	<0.001

© 2021 Chen ZH et al. *JAMA Network Open.*

eTable 6 cont.

Mediators	Melanoma				Bladder				Kidney			
	Female		Male		Female		Male		Female		Male	
	Effect size (95%CI)	*P*	Effect size (95%CI)	*P*	Effect size (95%CI)	*P*	Effect size (95%CI)	*P*	Effect size (95%CI)	*P*	Effect size (95%CI)	*P*
**Radiotherapy**												
**TE**	0.299 (0.215,0.383)	<0.001	0.349 (0.291,0.408)	<0.001	0.347 (0.259,0.435)	<0.001	0.411 (0.363,0.459)	<0.001	0.16 (0.116,0.203)	<0.001	0.162 (0.116,0.209)	<0.001
**NIE**	0 (-0.002,0.002)	0.81	0 (0,0.001)	0.296	0.009 (0.004,0.014)	<0.001	0.009 (0.006,0.011)	<0.001	0 (- 0.002,0.002)	<0.001	0 (0,0)	0.487
**NDE**	0.299 (0.215,0.383)	<0.001	0.349 (0.291,0.407)	<0.001	0.338 (0.25,0.426)	<0.001	0.402 (0.354,0.45)	<0.001	0.16 (0.116,0.203)	<0.001	0.162 (0.116,0.209)	<0.001
**PM**	0.001 (-0.005,0.007)	0.745	0.001 (-0.003,0.005)	0.528	0.031 (0.024,0.037)	<0.001	0.026 (0.023,0.029)	<0.001	-0.001 (- 0.005,0.003)	<0.001	-0.001 (-0.005,0.003)	0.695
**Chemotherapy**												
**TE**	0.299 (0.215,0.383)	<0.001	0.349 (0.291,0.407)	<0.001	0.343 (0.255,0.431)	<0.001	0.403 (0.355,0.451)	<0.001	0.159 (0.115,0.203)	<0.001	0.16 (0.113,0.206)	<0.001
**NIE**	0 (-0.001,0)	0.7135	0 (0,0)	0.657	0.005 (0,0.01)	0.047	0.001 (-0.001,0.003)	0.412	-0.001 (-0.001,0)	0.076	-0.003 (-0.003,- 0.002)	<0.001
**NDE**	0.299 (0.215,0.383)	<0.001	0.349 (0.291,0.407)	<0.001	0.338 (0.25,0.426)	<0.001	0.402 (0.354,0.45)	<0.001	0.16 (0.116,0.203)	<0.001	0.162 (0.116,0.209)	<0.001
**PM**	-0.001 (-0.007,0.005)	0.699	0 (-0.004,0.004)	0.88	0.017 (0.01,0.023)	<0.001	0.002 (-0.001,0.005)	0.139	-0.004 (-0.008,0)	0.059	-0.018 (-0.022,- 0.014)	<0.001

© 2021 Chen ZH et al. *JAMA Network Open.*

eTable 6 cont.

Mediators	Pancreas				Breast			
	Female		Male		Female		Male	
	Effect size (95%CI)	*P*	Effect size (95%CI)	*P*	Effect size (95%CI)	*P*	Effect size (95%CI)	*P*
**Stage**								
**TE**[a]	0.106 (0.074,0.138)	<0.001	0.213 (0.179,0.247)	<0.001	0.271 (0.25,0.293)	<0.001	0.546 (0.344,0.748)	0.003
**NIE**[b]	-0.012 (-0.025,0)	<0.001	-0.002 (-0.016,0.012)	<0.001	0.028 (0.026,0.03)	<0.001	0.032 (0,0.064)	0.277
**NDE**[c]	0.119 (0.089,0.148)	<0.001	0.215 (0.184,0.246)	<0.001	0.244 (0.223,0.265)	<0.001	0.514 (0.314,0.713)	0.005
**PM**[d]	-0.123 (-0.128, -0.118)	<0.001	-0.011 (-0.016,-0.006)	<0.001	0.115 (0.113,0.117)	<0.001	0.076 (0.061,0.09)	<0.001
**Surgery**								
**TE**	0.109 (0.078,0.141)	<0.001	0.188 (0.156,0.221)	<0.001	0.103 (0.07,0.135)	<0.001	0.567 (0.175,0.959)	0.005
**NIE**	0.035 (0.021,0.049)	<0.001	0.042 (0.029,0.056)	<0.001	0.004 (0.003,0.005)	<0.001	0 (-0.006,0.007)	0.899
**NDE**	0.074 (0.046,0.102)	<0.001	0.146 (0.116,0.175)	<0.001	0.098 (0.066,0.131)	<0.001	0.567 (0.175,0.958)	0.005
**PM**	0.331 (0.326,0.336)	<0.001	0.242 (0.238,0.247)	<0.001	0.042 (0.038,0.045)	<0.001	0.001 (-0.017,0.019)	0.914

© 2021 Chen ZH et al. *JAMA Network Open.*

eTable 6 cont.

Mediators	Pancreas				Breast			
	Female		Male		Female		Male	
	Effect size (95%CI)	*P*	Effect size (95%CI)	*P*	Effect size (95%CI)	*P*	Effect size (95%CI)	*P*
**Radiotherapy**								
**TE**	0.074 (0.046,0.102)	<0.001	0.147 (0.117,0.176)	<0.001	0.1 (0.067,0.132)	<0.001	0.567 (0.175,0.958)	0.005
**NIE**	0 (-0.001,0.001)	0.71	0.001 (0,0.002)	0.109	0.001 (0.001,0.002)	0.06731 2613	0 (- 0.001,0.001)	0.995
**NDE**	0.074 (0.046,0.102)	<0.001	0.146 (0.116,0.175)	<0.001	0.098 (0.066,0.131)	<0.001	0.567 (0.175,0.958)	0.005
**PM**	-0.003 (-0.006,0)	0.036	0.008 (0.005,0.011)	<0.001	0.014 (0.011,0.018)	<0.001	0 (- 0.018,0.018)	0.999
**Chemotherapy**								
**TE**	0.112 (0.083,0.14)	<0.001	0.191 (0.161,0.22)	<0.001	0.098 (0.065,0.13)	<0.001	0.576 (0.183,0.969)	0.004
**NIE**	0.037 (0.032,0.043)	<0.001	0.045 (0.039,0.051)	<0.001	-0.001 (- 0.002,0)	<0.001	0.009 (- 0.026,0.045)	0.604
**NDE**	0.074 (0.046,0.102)	<0.001	0.146 (0.116,0.175)	<0.001	0.098 (0.066,0.131)	<0.001	0.567 (0.175,0.958)	0.005
**PM**	0.345 (0.342,0.348)	<0.001	0.252 (0.249,0.256)	<0.001	-0.009 (- 0.013,-0.006)	<0.001	0.021 (0,0.043)	0.054

[a]Total effect; [b]Natural indirect effect; [c]Natural direct effect; [d]Proportion mediated

© 2021 Chen ZH et al. *JAMA Network Open.*

eTable 7. Demographic and Clinical Characteristics of Individuals Included and Excluded

Characteristics	Included 1733906 (81.3)	Excluded 397632 (18.7)	*P*
**Age at diagnosis**[a]	63.76 (12.60)	67.79 (14.90)	<0.001
**Race**[b]			<0.001
**White**	1393206 (80.4)	304445 (76.6)	
**Black**	201295 (11.6)	42183 (10.6)	
**Other**[c]	127420 (7.3)	23254 (5.8)	
**Unknown**	11985 (0.7)	27750 (7.0)	
**Sex**[b]			<0.001
**Female**	894379 (51.6)	163721 (41.2)	
**Male**	839527 (48.4)	233911 (58.8)	
**Grade**[b]			<0.001
**Grade I**	206578 (11.9)	34179 (8.6)	
**Grade II**	612491 (35.3)	92795 (23.3)	
**Grade III**	488671 (28.2)	67334 (16.9)	
**Grade IV**	60788 (3.5)	7827 (2.0)	
**Unknown**	365378 (21.1)	195497 (49.2)	
**Surgery**[b]			<0.001
**No**	536914 (31.0)	182464 (45.9)	
**Yes**	1196992 (69.0)	154303 (38.8)	
**Unknown**	0 (0.0)	60865 (15.3)	
**Radiation**[b]			<0.001
**No/ Unknown**	1159441 (66.9)	341790 (86.0)	
**Yes**	574465 (33.1)	55842 (14.0)	
**Chemotherapy**[b]			<0.001
**No/ Unknown**	1207743 (69.7)	350252 (88.1)	
**Yes**	526163 (30.3)	47380 (11.9)	

© 2021 Chen ZH et al. *JAMA Network Open.*

eTable 7 cont.

Characteristics	Included 1733906 (81.3)	Excluded 397632 (18.7)	*P*
**Stage**[b]			<0.001
**Early**	1189957 (68.6)	193049 (48.5)	
**Advanced**	543949 (31.4)	48769 (12.3)	
**Unknown**	0 (0.0)	155814 (39.2)	
**Insurance**[b]			<0.001
**Insured**	1475410 (85.1)	199736 (50.2)	
**Medicaid**	181047 (10.4)	27051 (6.8)	
**Uninsured**	39962 (2.3)	6337 (1.6)	
**Unknown**	37487 (2.2)	164508 (41.4)	
**Poverty**[a]	32.40 (9.55)	32.47 (9.62)	<0.001
**Education**[a]	14.87 (6.17)	14.76 (6.15)	<0.001
**Residence**[b]			<0.001
**Metropolitan**	1531155 (88.3)	350231 (88.1)	
**Nonmetropolitan**	201258 (11.6)	46226 (11.6)	
**Unknown**	1493 (0.1)	1175 (0.3)	

[a]Mean (SD); [b]N (%) includes American Indian, Alaska Native, and Asian/Pacific Islander

© 2021 Chen ZH et al. *JAMA Network Open.*
